# Human prolyl hydroxylase domain 2 reacts with O_2_ and 2-oxoglutarate to enable formation of inactive Fe(III).2OG.hypoxia-inducible-factor α complexes

**DOI:** 10.1038/s41598-024-75761-y

**Published:** 2024-10-30

**Authors:** Giorgia Fiorini, Stephen A. Marshall, William D. Figg, William K. Myers, Lennart Brewitz, Christopher J. Schofield

**Affiliations:** 1 Chemistry Research Laboratory, Department of Chemistry and the Ineos Oxford Institute for Antimicrobial Research, University of Oxford, 12 Mansfield Road, Oxford, OX1 3TA UK; 2 Inorganic Chemistry Laboratory, Department of Chemistry, South Parks Road, Oxford, OX1 3QR UK

**Keywords:** Hypoxia/oxygen sensing, Hypoxia inducible factor, Prolyl hydroxylase domain (PHD)/EGLN enzyme, α-Ketoglutarate/2-oxoglutarate oxygenase, L-Ascorbic acid/vitamin C, Epigenetics, Transcriptional regulation, Biochemistry, Chemical biology

## Abstract

Hypoxia inducible transcription factors (HIFs) mediate the hypoxic response in metazoans. When sufficient O_2_ is present, Fe(II)/2-oxoglutarate (2OG)-dependent oxygenases (human PHD1-3) promote HIFα degradation via prolyl-hydroxylation. We report crystallographic, spectroscopic, and biochemical characterization of stable and inactive PHD2.Fe(III).2OG complexes. Aerobic incubation of PHD2 with Fe(II) and 2OG enables formation of PHD2.Fe(III).2OG complexes which bind HIF1-2α to give inactive PHD2.Fe(III).2OG.HIF1-2α complexes. The Fe(III) oxidation state in the inactive complexes was shown by EPR spectroscopy. L-Ascorbate hinders formation of the PHD2.Fe(III).2OG.(+/-HIFα) complexes and slowly regenerates them to give the catalytically active PHD2.Fe(II).2OG complex. Crystallographic comparison of the PHD2.Fe(III).2OG.HIF2α complex with the analogous anaerobic Fe(II) complex reveals near identical structures. Exposure of the anaerobic PHD2.Fe(II).2OG.HIF2α crystals to O_2_ enables *in crystallo* hydroxylation. The resulting PHD2.product structure, manifests conformational changes compared to the substrate structures. The results have implications for the role of the PHDs in hypoxia sensing and open new opportunities for inhibition of the PHDs and other 2OG dependent oxygenases by promoting formation of stable Fe(III) complexes.

**Supplementary Information:**

The online version contains supplementary material available at 10.1038/s41598-024-75761-y.

## Introduction

The α,β -heterodimeric hypoxia inducible factors (HIFs) play key roles in the response to chronic hypoxia in humans and other animals. HIFα, but not HIFβ, levels are strongly regulated by O_2_ availability^1–3^: under hypoxic conditions HIFα levels increase and it dimerises with HIFβ, forming transcriptionally active α,β-HIF that binds to hypoxia response elements. α,β-HIF promotes the context dependent expression of genes, including those encoding for vascular endothelial growth factor (VEGF) and erythropoietin (EPO) which work to ameliorate the effects of hypoxia^3,4^.

**Fig. 1:**
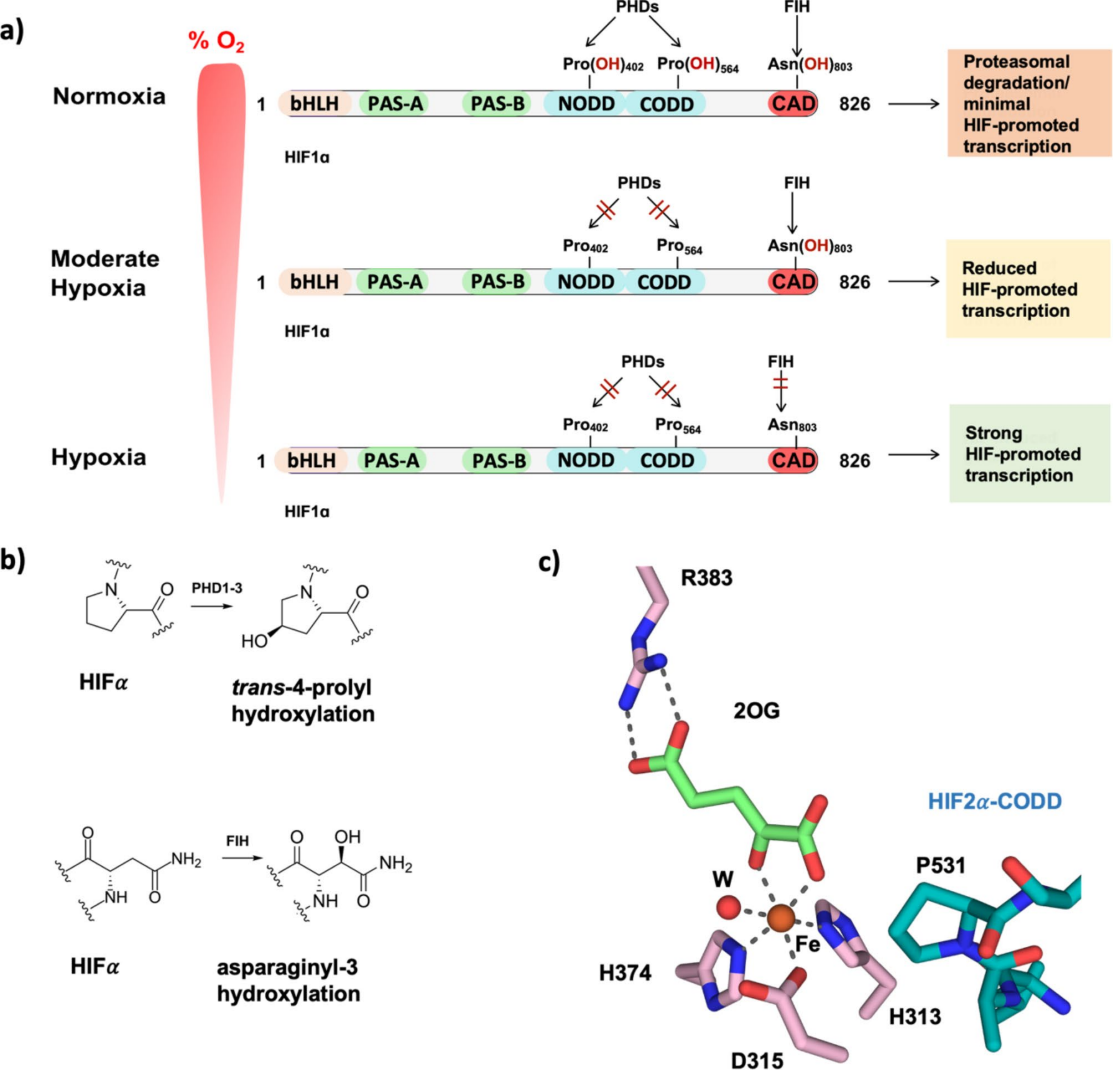
Overview of the roles of 2OG dependent oxygenases in the HIF mediated hypoxia response pathway. (a) PHD1-3 catalysed HIF1-3α prolyl-hydroxylation promotes proteasomal HIFα degradation. (b) PHD1-3 catalysed HIF1-3α *trans*-4-prolyl hydroxylation. FIH catalyses HIF1-2α. asparaginyl-3-hydroxylation. (c) The PHD2 active site (PDB: 8Q6D) with Fe(II) (orange sphere), 2OG (green), the chelating water (red) and HIF2α-CODD_523 − 542_ (teal). Note the C4 *endo*-conformation of Pro-531. Polar interactions: black dashes. bHLH: basic helix-loop-helix domain, PAS: Per-Arnt-Sim domain, CODD: C-terminal oxygen-dependent degradation domain, NODD: N-terminal oxygen-dependent degradation domain, CAD: C-terminal transcriptional activation domain. The figure was adapted from Fiorini and Schofield, 2024^39^.

Two types of 2-oxoglutarate (2OG) and Fe(II) dependent oxygenases (OGO) have roles in regulating the levels of HIFα isoforms and the transcriptional activity of α,β-HIF (Fig. 1a)^1,5–7^. In cells, the HIFα prolyl hydroxylases (PHDs) catalyse hydroxylation of prolyl-residues located in N- and C-terminal oxygen dependent degradation domains (NODD and CODD) of HIF1-2α, and the CODD of HIF3α. There are three human PHDs (PHD1-3) of which PHD2, which is characterized by the presence of a Zn-finger domain which recruits PHD2 to the translational machinery^8^, is highly conserved^9,10^. Prolyl-residue hydroxylation promotes binding of HIFα to the von Hippel-Lindau (VHL) ubiquitin ligase complex^6,11–14^, so targeting it for proteasomal degradation (Fig. 1a, b)^11–15^. FIH, which belongs to a different structural class of OGO than that of the PHDs^1,6,7,16^, catalyses C-3 hydroxylation of an asparagine-residue located in the C-terminal transcriptional activation domain (CAD) of HIF1-2α (HIF3α lacks a CAD domain)^17^; CAD hydroxylation hinders binding of HIF1-2α to the histone acetyl transferases CBP/p300 (Fig. 1a, b)^18^.

Whilst there is clear evidence that FIH has multiple substrates other than HIFα^19,20^, the situation is less clear for the PHDs, with conflicting biochemical and cellular reports^21^. Indeed, although the ‘biochemical’ roles of the PHDs and FIH in the hypoxic response may appear relatively simple, many molecular details of the hypoxic response are poorly understood. These include how context dependent regulation of HIF target genes is achieved, how the O_2_ dependency of the HIF hydroxylases is linked with other redox variables, and how the PHDs and FIH enable efficient modifications of the low levels of HIFα normally present in cells in normoxic conditions.

At the resting enzyme stage in the consensus OGO mechanism (Fig. S1)^22–24^, the active site Fe(II) is complexed by two histidine- and, typically, an aspartate-or glutamate-residue and 2–3 waters. 2OG then binds to the Fe(II) in a bidentate manner, via its C-1 carboxylate and C-2 ketone oxygen. The substrate then binds in a manner that, at least sometimes, promotes loss of an Fe(II) complexed water so opening a vacant coordination site for O_2_ binding^22–26^. Oxidative decarboxylation then occurs (Fig. S1) giving succinate and an Fe(IV)= O species^22–24,27^; the latter effects substrate oxidation, typically hydroxylation. Mechanistic variations occur, including in the rates of individual steps, in some cases enabling observation of an Fe(IV) =O intermediate^22–24,28,29^. Modelling and experimental studies provide evidence that active site events during OGO catalysis can be coupled to motions in the second coordination sphere and beyond^30,31^.

It is proposed that the kinetic properties of PHDs reflect their hypoxia sensing role—they manifest relatively high O_2_ K_M_ values (65 to >450 µM) ^33–39^ and slow reactions of PHD2.Fe(II).2OG.HIFα complexes with O_2_ have been observed^34–36^. There is evidence that PHD2 forms an unusually stable complex with Fe and 2OG^35,37^. Modelling studies also suggest that the crystallographically observed 2OG carboxylate coordination mode may change during PHD2 catalysis, that is from a bidentate to a monodentate coordination mode in order to enable O_2_ binding^38^ (Fig. S1).

In the absence of substrates, many OGO catalyse the reaction of 2OG and O_2_ to give succinate and CO_2_, though the capacity for this varies^16,22,23,40,41^. Since conversion of 2OG and O_2_ to succinate and CO_2_ is a two-electron oxidation, reduction is required to complete ‘uncoupled’ reaction cycles if they occur catalytically. With the PHDs, reducing agents that promote catalysis include glutathione and L-ascorbate (L-Asc/vitamin C)^33,40,42,43^.

Given the roles of many human OGO in disease, there is interest in their relationships with redox active agents, especially L-Asc, administration of which has been evaluated for treatment of diseases including cancer^44,45^. Lack of L-Asc correlates with the disease scurvy, symptoms of which are, at least in part, likely due to impaired collagen biosynthesis associated with reduced procollagen prolyl hydroxylase activity^46^. Multiple studies with proteins and cells have reported on positive effects of reducing agents on activities of OGO involved in biologically important processes^42,43,47^, including lipid metabolism, DNA repair and chromatin modifications^48^. The mechanism(s) underlying the apparent L-Asc mediated increases in OGO activity in cells are unclear. Further, although OGO are involved in biosynthesis of societally important molecules, e.g. β-lactam and other antibiotics^49,50^, their redox (and conformational) sensitivity means their potential as isolated biocatalysts is largely unexploited^51^.

Here we report spectroscopic, kinetic, and crystallographic studies demonstrating PHD2 undergoes reaction with 2OG and O_2_ to give a stable PHD2.Fe(III).2OG complex that binds HIFα isoforms, giving catalytically inert complexes. The presence of L-Asc both hinders formation of PHD2.Fe(III).2OG complexes and promotes their reactivation, possibly through reduction of Fe(III) to Fe(II) to give PHD2.Fe(II).2OG complexes. The results indicate potential for a new interface between OGO and redox biochemistry and suggest new ways to modulate OGO activity.

## Results

### PHD2 forms inactive Fe(III).2OG and Fe(III).2OG.substrate complexes

**Fig. 2:**
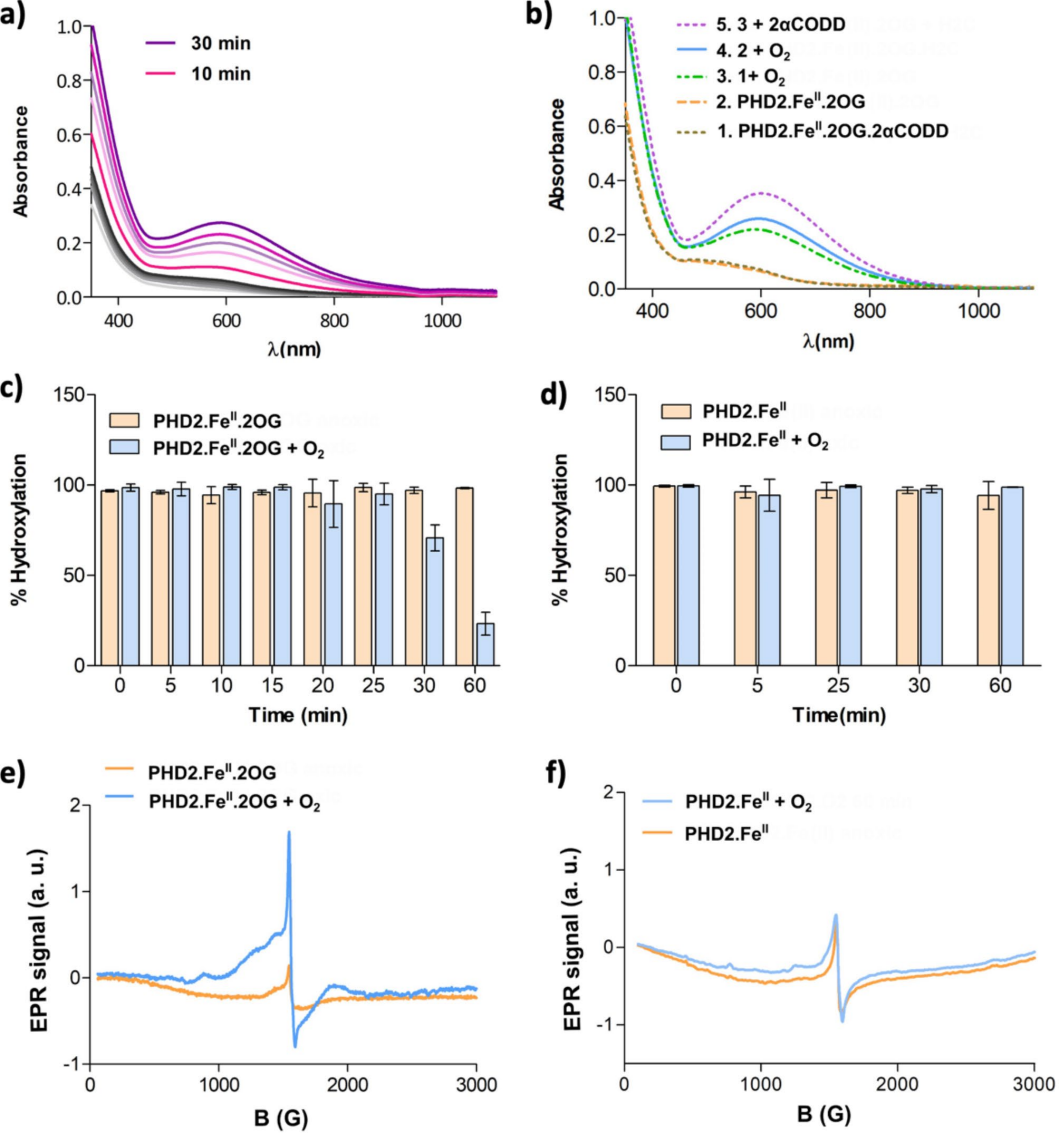
Spectroscopic studies showing formation of a catalytically inactive PHD2.Fe(III).2OG complex with a blue chromophore. (a) UV-vis spectra of an O_2_ exposed mixture of PHD2 (300 µM), (NH_4_)_2_Fe(II)(SO_4_) (250 µM), 2OG (3 mM) and HIF2α-CODD (250 µM). Grey-scale plots show 1 min intervals up to 10 min. Spectra between 10 and 30 min are in 5 min intervals. (b) UV-vis spectra of anaerobic and an O_2_ exposed mixture of PHD2 (300 µM), (NH_4_)_2_Fe(II)(SO_4_) (250 µM), 2OG (3 mM), HIF2α-CODD (250 µM if present). For spectra 3 and 4, the samples were exposed to O_2_ for 60 min. (c) The extent of hydroxylation when a PHD2.Fe(II).2OG mixture is added to an HIF1α-CODD_556 − 574_ solution, with or without prior O_2_ exposure of the PHD2.Fe(II).2OG mixture. A clear decrease in hydroxylation is observed for the O_2_ exposed mixture after ~ 30 and 60 min. No activity loss was observed for the anaerobic control. The results are means of 3 independent assays (*n* = 3; mean ± SD). (d) Comparison of hydroxylation observed when a PHD2.Fe(II) mixture is added to HIF1α-CODD_556 − 574_ and 2OG, both with and without prior O_2_ exposure of the PHD2.Fe(II) mixture. No activity loss was observed for the anaerobic control and the O_2_ exposed sample. The results are means of 3 independent runs (*n* = 3; mean ± SD). (e) EPR spectra of anaerobic PHD2.Fe(II).2OG (orange line) and O_2_ exposed (blue line, 60 min) PHD2.Fe(II).2OG. (f) EPR spectra of anaerobic PHD2.Fe(II) (orange line) and O_2_ exposed (blue line, 60 min) PHD2.Fe(II).

Assays to investigate PHD2 kinetics/investigate inhibitors are typically carried out with an excess of co-substrates/substrate^31,34,52^. In studies aimed at characterising intermediates, we employed conditions employing an excess of the catalytic domain of PHD2_181 − 407_ (hereafter referred to as PHD2) relative to Fe(II) and substrate to ensure optimal binding of the latter two components^34,36^. We observed that exposure of an anaerobic mixture of 300 µM PHD2, 250 µM Fe(II), 3 mM 2OG and 250 µM HIF2α-CODD to O_2_ leads to formation of a blue-coloured solution with a λ_max_ of 603 nm (Fig. 2a, b-green-dashed line). By contrast, the UV-vis spectrum of the corresponding anaerobic sample shows a λ_max_ in the 520 nm region, consistent with the formation of a PHD2.Fe(II).2OG.CODD complex (Fig. 2a, b-grey dashed line)^36^. The aerobic λ_max_ 603 nm chromophore was observed to form faster at 20 °C than at 4 °C (Fig. S3).

To investigate the nature of the new PHD2 chromophore and its dependence on the presence of HIF2α-CODD, an anaerobic mixture of 300 µM PHD2, 250 µM Fe(II), 3 mM 2OG was exposed to O_2_ and monitored over time by UV-vis spectroscopy. After ~ 10 min of O_2_ exposure, the peak intensity in the 520 nm region was observed to decrease in a manner concomitant with the formation of a new feature in the 596 nm region, which reached a maximum after 60 min (Fig. 2b-blue line). Addition of HIF2α-CODD or HIF1α-CODD to the PHD2, Fe(II) and O_2_ derived chromophore caused a small peak shift from 596 nm to 603 nm (Figs. 2b-purple dashed line, S2).

The catalytic activity of the O_2_ derived chromophore was investigated using mass spectrometry (MS): an anaerobic mixture of 300 µM PHD2, 250 µM Fe(II) and 3 mM 2OG was exposed to O_2_ for 60 min, a time when the mixture was visibly blue, then added into aerobic buffer containing 10 µM HIF1α-CODD (with 100 fold dilution). After 30 min the reaction was quenched with 0.1%_(v/v)_ formic acid. A control in which the PHD2.Fe(II).2OG mixture (1:0.8:10 ratio) was kept anaerobic for 60 min prior to incubation with HIF1α-CODD in O_2_ exposed buffer was carried out. The PHD2.Fe(II).HIF1α-CODD mixture was not blue coloured when kept anaerobic. Notably, and reproducibly, only ~ 18% HIF1α-CODD was hydroxylated by the O_2_ exposed mixture, whilst ~ 90% HIF1α-CODD was hydroxylated by a sample that was kept anaerobic prior to O_2_ exposure (Fig. 2c). These results imply formation of the ~ 600 nm chromophore requires O_2_ and is linked to loss of PHD2 activity.

Loss of activity in OGO can occur as a result of 2OG promoted oxidation of Fe(II) to Fe(III) (or, potentially a higher oxidation state), occurring either in solution or at the active site^21,40,51^. To investigate the role of 2OG in the loss of PHD2 activity, we conducted the same assay as described for the PHD2.Fe(II).2OG mixture, but without 2OG. An anaerobic solution containing 300 µM PHD2 and 250 µM Fe(II) was exposed to O_2_; activity was then monitored over time by MS, by incubating the PHD2.Fe(II) mixture (1:100 dilution) with 10 µM HIF1α-CODD and 300 µM 2OG for 30 min prior to quenching. The same experiment was conducted on a PHD2.Fe(II) mixture (1:0.8 ratio) that was kept anaerobic. Interestingly, neither condition showed loss of activity, indicating that, in addition to O_2_, the presence of 2OG promotes loss of activity (in the absence of substrate) (Fig. 2d).

To investigate the Fe-oxidation state of the inactive species, we conducted electron paramagnetic resonance (EPR) studies on an anaerobic PHD2.Fe.2OG mixture (1:0.8:10 ratio) and a (blue) sample obtained by exposing the same mixture to O_2_ for 60 min. The spectrum of the anaerobic sample manifested a low intensity peak at g_//_ = 4.2 indicating a low level of Fe(III), possibly present in solution^37^. A g_//_ = 4.2 signal was observed with the blue sample, but with substantially higher intensity (Fig. 2e), suggesting that Fe(III) is likely the most abundant species present. When the PHD2.Fe(II) mixture was exposed to O_2_ in the absence of 2OG, the intensity of the Fe(III) signal did not show a substantial increase after O_2_ exposure (Fig. 2f), apparently reflecting the relative lack of inactivation without 2OG (Fig. 2d).

To investigate whether the inactive complex arises from the interaction of PHD2 with Fe(III) and 2OG, we measured the UV-vis spectra of a mixture of 300 µM PHD2 and 250 µM Fe(III), with and without 3 mM 2OG (Fig. S4). The resulting spectra revealed the formation of a chromophore with λ_max_= 596 nm within 5 min on mixing PHD2 with Fe(III) and 2OG, both in anaerobic (Fig. S4 orange-dashed line) and aerobic environments (Fig. S4, blue line). By contrast no λ_max_ ~600 nm peak was observed to form in the absence of 2OG (Fig. S4, green-dashed line). These results suggest that the λ_max_ ~600 nm peak is a consequence of formation of a PHD2.Fe(III).2OG complex.

Studies on other non-heme Fe(II) oxyenases including, taurine dioxygenase (TauD)^53^, TfdA^54^ and FIH^51^ have reported on the observation of a blue colour as a result of auto-hydroxylation of residues near the active site. To investigate whether self-hydroxylation of PHD2 is responsible for the appearance of the observed blue colour/λ_max_ ~600 nm peak, we collected MS spectra of a blue solution of PHD2.Fe.2OG (obtained exposing PHD2.Fe(II).2OG to O_2_, as described in the Supplementary Methods) (Fig. S5). Mass spectra of apo-PHD2 and of an anaerobic mixture of PHD2.Fe(II).2OG were collected (Fig. S5). The results imply that the mass of PHD2 protein is unchanged after formation of the blue complex.

### Crystal structure of the PHD2.Fe(II)/Fe(III).2OG.substrate and PHD2.product complexes

**Fig. 3:**
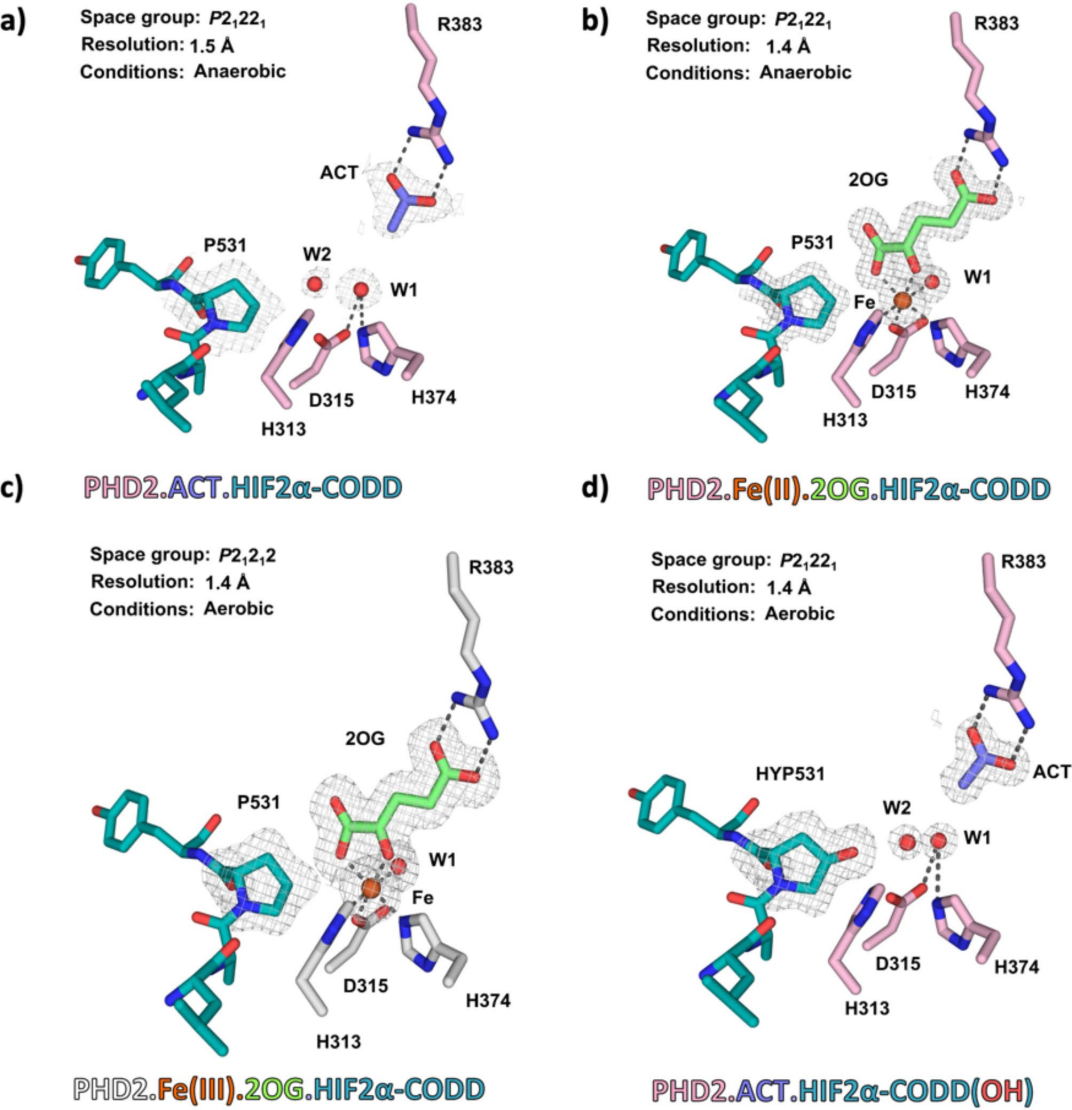
Comparison of crystal structure views of PHD2.Fe(II)/Fe(III).2OG.HIF2α-CODD/HIF2α-CODD(OH) complexes. (a) View of the electron density map (Polder map, carved to 3σ) of the acetate (ACT) (purple sticks), water (red spheres), and LAPY residues from HIF2α-CODD (teal sticks) in the PHD2.ACT.HIF2α-CODD complex (PDB: 8Q5S). (b) View of the electron density map (Polder map, carved to 3σ) of 2OG (green sticks), Fe (orange), water, and LAPY residues from HIF2α-CODD (teal sticks) in the PHD2.Fe(II).2OG.HIF2α-CODD complex (PDB: 8Q6D) coloured as in A. (c) View of the electron density map (Polder map, carved to 3σ) of 2OG (green), Fe, water and LAPY residues from HIF2α-CODD (shown as teal sticks) in the PHD2.Fe(III).2OG.HIF2α-CODD complex (PDB: 8Q6E). (d) A view of the electron density (Polder map, carved to 3σ) of ACT, water, and LAPY residues from hydroxylated HIF2α-CODD in the PHD2.ACT.HIF2α-CODD(OH) complex (PDB: 8Q64); coloured as in A. Note the retention of the C4-*endo* conformation in both substrate and product complex structures.

Attempts were made to structurally characterize the inactive chromophores that PHD2 forms with Fe(III) and HIF1-2α-CODD. Crystal structures of PHD2_181 − 407_ and PHD2_181 − 426_ in complex with inhibitors or HIF1-2α ODDs are reported^52,55–58^, however, there are no reported structures of catalytically active PHD2.Fe.2OG.ODD complexes. Following screening of PHD2_181-407_, Fe(II), 2OG and HIFαa-CODD (residues 523–542) in an anaerobic environment, plate morphology crystals diffracting to 1.5 Å resolution (*P*2_1_22_1_ space group) were obtained. Despite both Fe(II) and 2OG being present in the PHD2 HIF2α substrate mixture used for crystallisation, under these conditions no electron density was observed for a metal ion or co-substrate at the active site. Instead, an active site located acetate ion (ACT; arising from the crystallisation conditions) was observed giving a PHD2.ACT.HIF2α-CODD complex; the acetate ion is positioned to interact with the Arg383 guanidinium group, which interacts analogously with the 2OG C5 carboxylate^55^ (Fig. 3a). Use of increased concentrations of Fe(II) and 2OG in the protein solution, (see Supplementary Methods for details), resulted in thin plate morphology anaerobic crystals of PHD2.Fe(II).2OG.HIF2α-CODD, which diffracted to 1.4 Å resolution (*P*2_1_22_1_) (Fig. 3b). The same crystallisation conditions were used to obtain aerobic crystals from a blue coloured mixture, containing PHD2, Fe(III), 2OG and HIF2α-CODD, diffracting to 1.4 Å resolution (*P*2_1_2_1_2) revealing a PHD2.Fe(III).2OG.HIF2α-CODD complex structure (Fig. 3c). Note, although the redox state of the Fe after diffraction of PHD2 crystals is uncertain, prior to diffraction it can be inferred as being Fe(III) from the results of the O_2_ exposure studies, showing the PHD2.Fe(III).2OG.HIF2α-CODD complex crystals to be catalytically inactive (see below).

Superimposition of the PHD2.Fe(II).2OG.HIF2α-CODD structure with the analogous Fe(III) complex structure shows that the two structures are strikingly similar (RMSD 0.056 Å), including with respect to the conformation of the β2-β3 loop and other PHD2:HIF2α-CODD interactions, which are similar to those reported^55^; note the importance of dynamic motions of the β2-β3 loop conformation in catalysis and determining substrate selectivity^32,55^ (Fig. S6a).

The active site structures are very similar in the PHD2.2OG.HIF2α-CODD complexes with Fe(II) or Fe(III) (Fig. 3b); in both cases Fe is coordinated by His313, His374, Asp315, a water (*trans* to His313) and 2OG (as a bidentate ligand). Comparison of the PHD2.Fe(II)/Fe(III).2OG.HIF2α-CODD structures with the PHD2.HIF2α-CODD structure shows the Fe-coordinating water is present in all three complexes (Fig. 3a–c). As reported in studies on PHD.HIFα substrate complexes, in all our structures, the pyrrolidine ring of Pro531_HIF2α − CODD_ is, at least predominantly, in the C4-*endo* conformation (Fig. S7a–c)^56^.

To investigate the catalytic activity of the PHD2.Fe(II).2OG.HIF2α-CODD complex, the complex crystals were exposed to O_2_ for 60 min, then cooled in liquid N_2_ and analysed by X-ray diffraction (1.4 Å resolution, *P*2_1_22_1_). The results revealed a PHD2.ACT.HIF2α-CODD(OH) product complex, in which Pro531_2α − CODD_ is clearly C4-hydroxylated (Fig. 3d), demonstrating the catalytically productive nature of the C4-*endo* prolyl conformation observed in the PHD2.Fe(II).2OG.HIF2α-CODD structure. Although superimposition of the product complex with the anaerobic PHD2.Fe(II).2OG.HIF2α-CODD complex reveals similar overall structures (RMSD of 0.071 Å) (Fig. S6b), there are changes at the active site. Interestingly, the Fe ion has exited the active site, apparently being replaced by a water molecule, which interacts with the sidechains of Asp315 and His374. An acetate ion is bound in the 2OG binding pocket, as observed in the PHD2.ACT.HIF2α-CODD structure (Fig. 3d).

The observation of the PHD2.ACT.HIF2α-CODD and PHD2.ACT.HIF2α-CODD(OH) structures lacking Fe and 2OG/succinate likely, at least in part,  reflects symmetry related crystallography interactions stabilising the PHD2.HIF2α complexes. Nonetheless, these structures raise the possibility that such apo-complexes may be of biological relevance, e.g., under conditions when either Fe or 2OG are limiting.

In the substrate binding groove of PHD2, the hydroxylated HIF2α-CODD product maintains all key interactions observed in the PHD2.substrate complexes^52,55,56^ (Figs. S6, 7). Interestingly, the active site complexed HyPro531_2α − CODD_ product apparently retains the C4-*endo* conformation (Fig. S7d), contrasting with observations in the previously reported structures of hydroxylated HIFα-CODD in complex with the von Hippel-Lindau protein and elongin B/C, where HyPro531_2α − CODD_ adopts the thermodynamically more favoured C4-*exo* conformation^59–61^.

Whilst O_2_ exposure of the anaerobic PHD2.Fe(II).2OG.HIF2α-CODD complex led to *in crystallo* hydroxylation, the lack of prolyl-hydroxylation in the analogous PHD2.Fe(III) complex, despite the crystals being obtained in the presence of O_2_, supports the assigned Fe(III) oxidation state and inactivity of the PHD2.Fe(III).2OG.HIF2α-CODD complex (Fig. 1c).

### Role of L-ascorbic acid in PHD2 activity regeneration and activity loss prevention

**Fig. 4:**
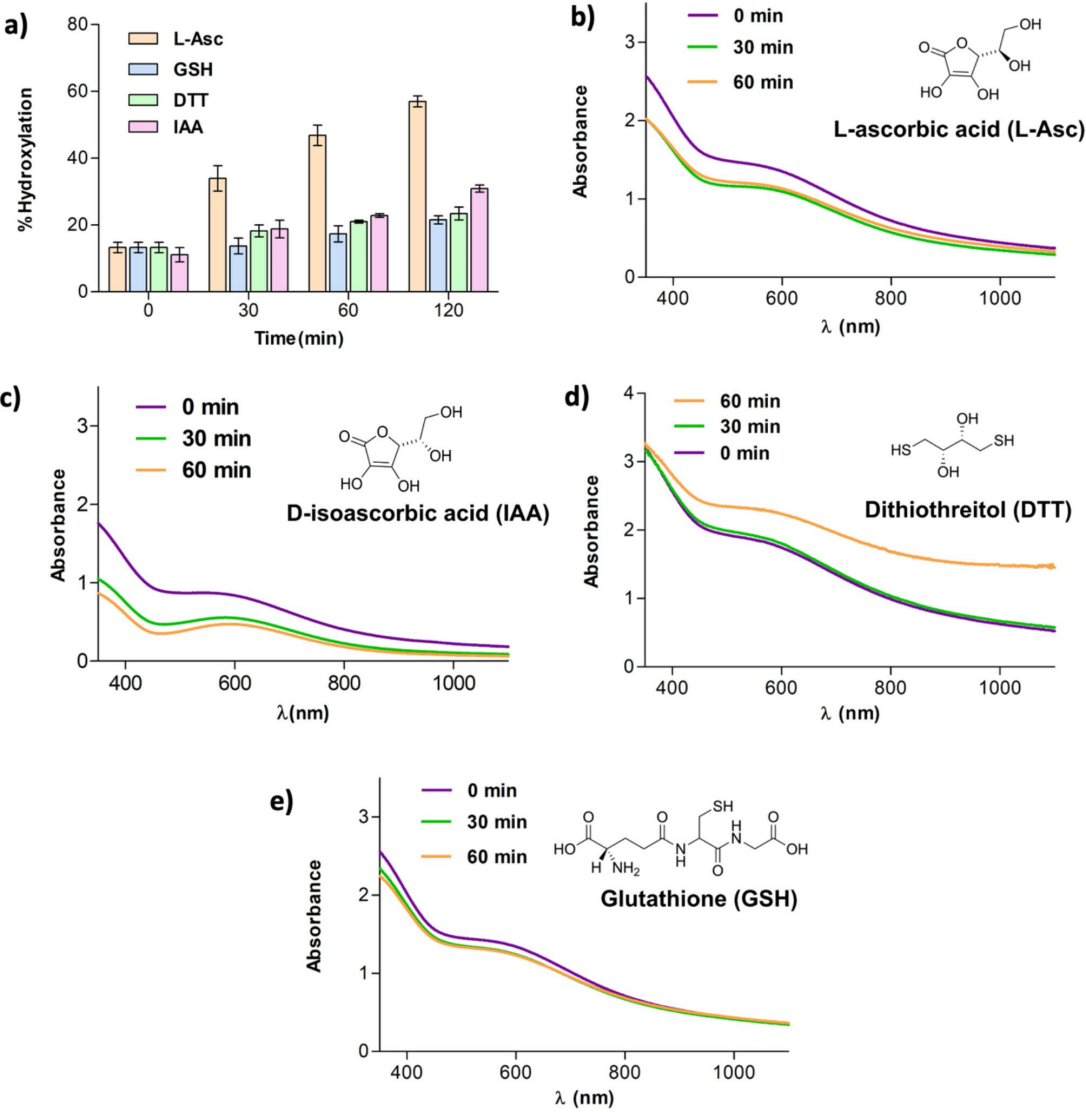
Hydroxylation assays and UV-vis spectra displaying the effects of addition of reducing agents on the blue PHD2.Fe(III).2OG complex. (a) The PHD2.Fe(III).2OG complex was obtained by exposing a mixture of PHD2 (300 µM), (NH_4_)_2_Fe(II)(SO_4_) (250 µM) and 2OG (3 mM) to O_2_ (60 min). HIF1α-CODD hydroxylation was monitored prior to and after incubation with L-Asc, GSH, DTT or IAA) (see Supplementary Methods for details). The extent of hydroxylation is displayed in the absence of reducing agents (bars at 0 min) and after incubation (for 30, 60 and 120 min) with 1 mM L-Asc (orange bars), 1 mM GSH (blue bars) and 1 mM DTT (dark green bars). Results are means of 3 independent replicates (*n* = 3, mean ± SD) (b) UV-vis spectra of the PHD2.Fe(III).2OG complex prior to (0 min, purple) and after reaction with 1 mM L-Asc for 30 min (green) and 60 min (orange). (c) UV-vis spectra of the PHD2.Fe(III).2OG complex prior to (0 min, purple), and after reaction with 1 mM IAA for 30 min (green) and 60 min (orange). (d) UV-vis spectra of the PHD2.Fe(III).2OG complex prior to (0 min-purple) and after treatment with 1 mM DTT for 30 min (green) and 60 min (orange). (E) UV-vis spectra of the PHD2.Fe(III).2OG complex prior to (0 min, purple), and after treatment with 1 mM GSH for 30 min (green) and 60 min (orange).

Ascorbic acid promotes catalysis by isolated PHD2, likely at least in part, via reduction of previously uncharacterized catalytically inert species^33,42^. We attempted to regenerate PHD2 activity after formation of the inactive PHD2.Fe(III).2OG complex by adding 1 mM sodium ascorbate (L-Asc) to the PHD2.Fe(III).2OG complex, obtained by exposing 300 µM PHD2, 250 µM Fe(II) and 3 mM 2OG to O_2_ for 60 min prior to L-Asc addition. UV-vis analysis after incubation with L-Asc manifested a time dependent decrease in the 596 nm absorbance, consistent with reduction of the Fe(III) complex. (Fig. 4b).

Other reducing agents of biological/assay interest were investigated, that is D-isoascorbic acid (IAA)^42^, glutathione (GSH) - due to its abundance in cells^62^, and dithiothreitol (DTT), which is commonly added to OGO assays^42^. As with L-Asc, incubation of IAA with the PHD2.Fe(III).2OG complex (prepared as previously described), caused a decrease in the absorbance at λ_max_ 596 nm (Fig. 4b, c). By contrast, incubation of GSH or DTT with the PHD2.Fe(III).2OG complex did not manifest clear effects on absorbance in the 600 nm region, indicating treatment with these thiols does not (efficiently) regenerate PHD2 activity (Fig. 4d, e).

MS experiments indicated that the decrease in absorbance observed with L-Asc reflects regeneration of catalytic activity. Studies were carried out to compare the activity of the PHD2.Fe(III).2OG complex, obtained by exposing the Fe(II) containing PHD2 to O_2_ for 60 min, with the same sample reacted with 1 mM L-Asc for 30 min prior to incubation with HIF1α-CODD. The results show the extent of HIF1α-CODD hydroxylation increased from ~ 13% to ~ 34% (~ 2.6 fold) when L-Asc was present prior to incubation with HIF1α-CODD (Fig. 4a). When the time of the reaction of the PHD2.Fe(III).2OG complex with L-Asc was increased from 30 min to 2 h, ~ 57% HIF1α-CODD hydroxylation was observed (Fig. 4a). When L-Asc was replaced with IAA, a substantial increase in activity was observed only after 2 h of reaction of IAA with the PHD2.Fe(III).2OG complex prior to incubation with HIF1α-CODD (~ 11% hydroxylation was observed without IAA and ~ 30% hydroxylation with IAA ). When L-Asc was replaced with GSH or DTT (1 mM), no substantial increase in activity was observed.

**Fig. 5:**
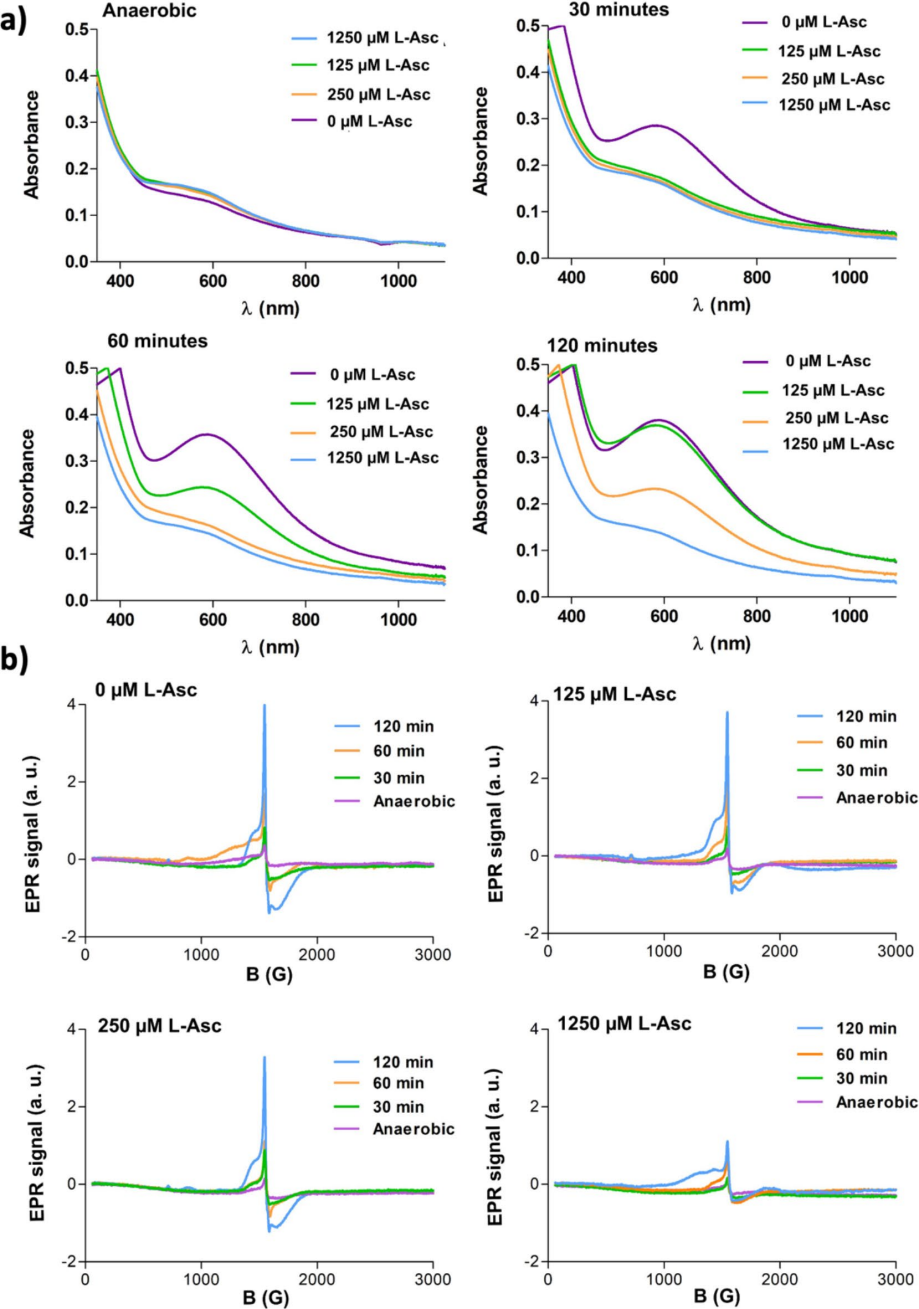
UV-vis and EPR spectroscopy data investigating the effect of L-ascorbate on the formation of the PHD2.Fe(III).2OG ∼600 nm absorbance. (a) UV-vis spectra of a PHD2_181 − 407_.Fe(II).2OG mixture containing increasing concentrations of L-Asc (0 µM-purple, 125 µM-green, 250 µM-orange and 1250 µM-light blue) which was exposed to O_2_ for 0, 30, 60, and 120 min. The extent of formation of the blue chromophore decreases with increasing L-Asc concentrations; no blue chromophore is observed with L-Asc at 1250 µM. (b) EPR analysis of a PHD2.Fe(II).2OG solution treated with increasing concentrations of L-Asc (0, 125, 250 and 1250 µM), then exposed to O_2_ for (0 min—purple; 30 min—green; 60 min—orange; and 120 min—light blue). Note that the intensity of the signal at g_//_=4.2 (which is indicative of an Fe(III) complex) is reduced at higher L-Asc concentrations.

Notably, when 1 mM L-Asc was added to a PHD2.Fe(II).2OG mixture (1:0.8:10 ratio) under anaerobic conditions, no loss of activity was observed after subsequent O_2_ exposure; thus, ~ 100% hydroxylation was observed for a 30-minutes O_2_ exposed mixture, ~ 40% more than when L-Asc was absent. These results imply the presence of sufficient L-Asc can avert PHD2 inactivation by preventing formation of the catalytically inert PHD2.Fe(III).2OG complex (Fig. 5a).

**Fig. 6:**
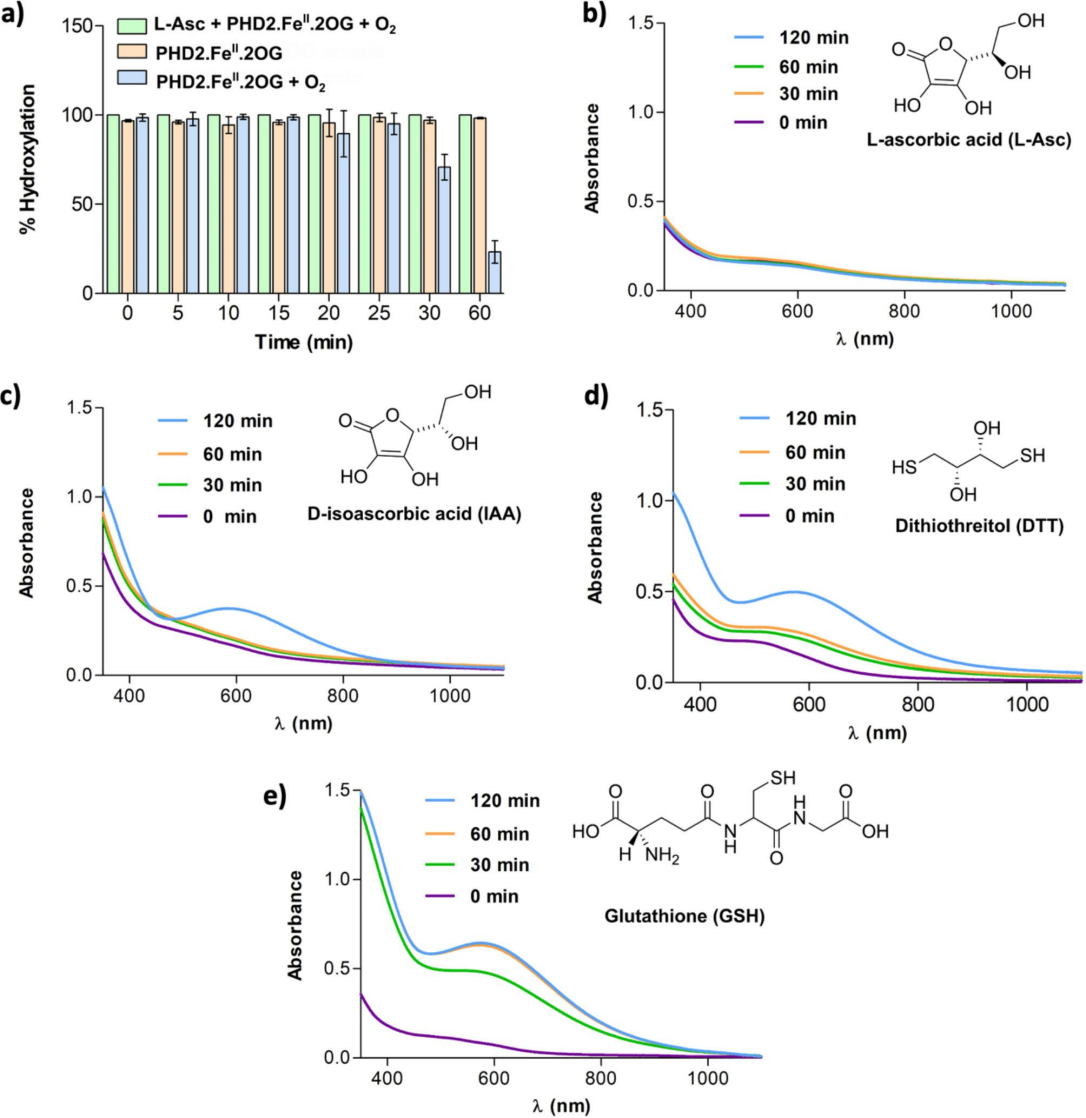
Hydroxylation assays and UV-vis spectra showing that L-Asc, but not thiol reducing agents, hinders formation of the ∼600 nm absorbance. (a) Comparison of hydroxylation observed when a L-Asc and PHD2.Fe(II).2OG mixture (green bars) is added to HIF1α-CODD_556 − 574_ either after O_2_ exposure (blue bars), or before O_2_ exposure (orange bars) (see Experimental Procedure for details). No activity loss was observed for the anaerobic and L-Asc containing samples (orange and green bars). The results are means of 3 independent runs (*n* = 3, mean ± SD). (b-e) UV-vis spectra of 1 mM L-Asc/D-isoascorbic acid/DTT/GSH and PHD2.Fe(II).2OG mixture analysed after 0 (purple), 30 (green), 60 (orange) and 120 min (light blue) of O_2_ exposure. The blue chromophore was not observed in the L-Asc containing sample. IAA was also observed to slow formation of the blue complex. No clear effect was observed with DTT. An increase in rate of formation of the blue chromophore was observed with GSH.

Reaction in the presence of L-Asc was observed to slow formation of the PHD2.Fe(III).2OG ∼600 nm absorbance resulting from exposure of PHD2.Fe(II).2OG (1:0.8:10 ratio) to O_2_. Anaerobic samples containing 300 µM PHD2, 250 µM Fe(II), 3 mM 2OG and L-Asc at different concentrations (0-1250 µM) were exposed to O_2_. UV-vis spectra were collected at 30, 60 and 120 min. (Fig. 6) Samples were frozen in liquid N_2_ immediately after UV-vis data collection for EPR studies. The UV-vis spectra revealed that formation of the ∼600 nm absorbance is slowed by the presence of increasing amounts of L-Asc, with no ∼600 nm peak being observed when L-Asc was present at 1250 µM (Figs. 6 and 5b). Similarly, EPR spectra of the UV-vis analysed samples revealed that the increase of intensity of the Fe(III) signal (g_//_ = 4.2) was slowed by increasing amount of L-Asc (Fig. 6b).

Previous studies have shown that D-isoascorbic acid (IAA) can partially replace L-Asc in promoting PHD2 maximal activity under standard turnover conditions^42^. When IAA was present at 1 mM, formation of the ~600 nm absorbance under aerobic conditions was slowed substantially (0.002 ± 0.002 abs/min versus 0.009 ± 0.001 abs/min when no IAA acid is present) (Fig. 5c). By contrast, the same amount of DTT had no substantial effect on the rate of formation of the ∼600 nm absorbance(0.009 ± 0.008 abs/min) (Fig. 5d). Interestingly, the presence of 1 mM GSH increased the rate of formation of the ∼600 nm absorbance from 0.009 ± 0.001 abs/min to 0.025 ± 0.004 abs/min (Fig. 5e). This effect is reflected in the relatively faster decrease in the activity of PHD2 when GSH was present in the PHD2.Fe(II).2OG assay mixture; when the PHD2.Fe(II).2OG complex was exposed to O_2_ for 25 min in the presence of 1 mM GSH, ~ 69% hydroxylation is observed, ~ 25% less than that observed without GSH (Fig. S14a, b).

Overall, L-Asc was found to be the most effective of the tested agents in preventing the activity loss due to PHD2.Fe(III).2OG formation and regenerating PHD2 activity. Interestingly GSH was observed to apparently promote PHD2 inactivation by an unknown mechanism.

Many OGO can catalyse the substrate uncoupled O_2_ dependent 2OG turnover to succinate and CO_2_. L-Asc is proposed to have a role in reducing catalytically inert species arising from this process^22,23^. Studies on procollagen prolyl-hydroxylase have shown that, at least in some cases, substrate 2OG uncoupled turnover is apparently coupled to stoichiometric L-Asc consumption^40^. 2OG can also react with hydrogen peroxide to give succinate^63^ To further investigate if L-Asc hinders ∼600 nm absorbance formation, we used ^1^H-NMR to monitor an O_2_ exposed mixture of 20 µM PHD2, 50 µM Fe(II), 1 mM 2OG, ± 4 mM L-Asc. We observed substantially more succinate formation in the L-Asc containing mixture, that is ∼150 µM succinate was formed after 22 min of O_2_ exposure compared to 30 µM succinate in the absence of L-Asc; the latter value is close to the measured amount of PHD2 present in the assay (20 µM) (Fig. S8). A control assay on the PHD2-free mixture confirmed that succinate formation from 2OG is enzyme dependent (Fig. S8).

**Fig. 7:**
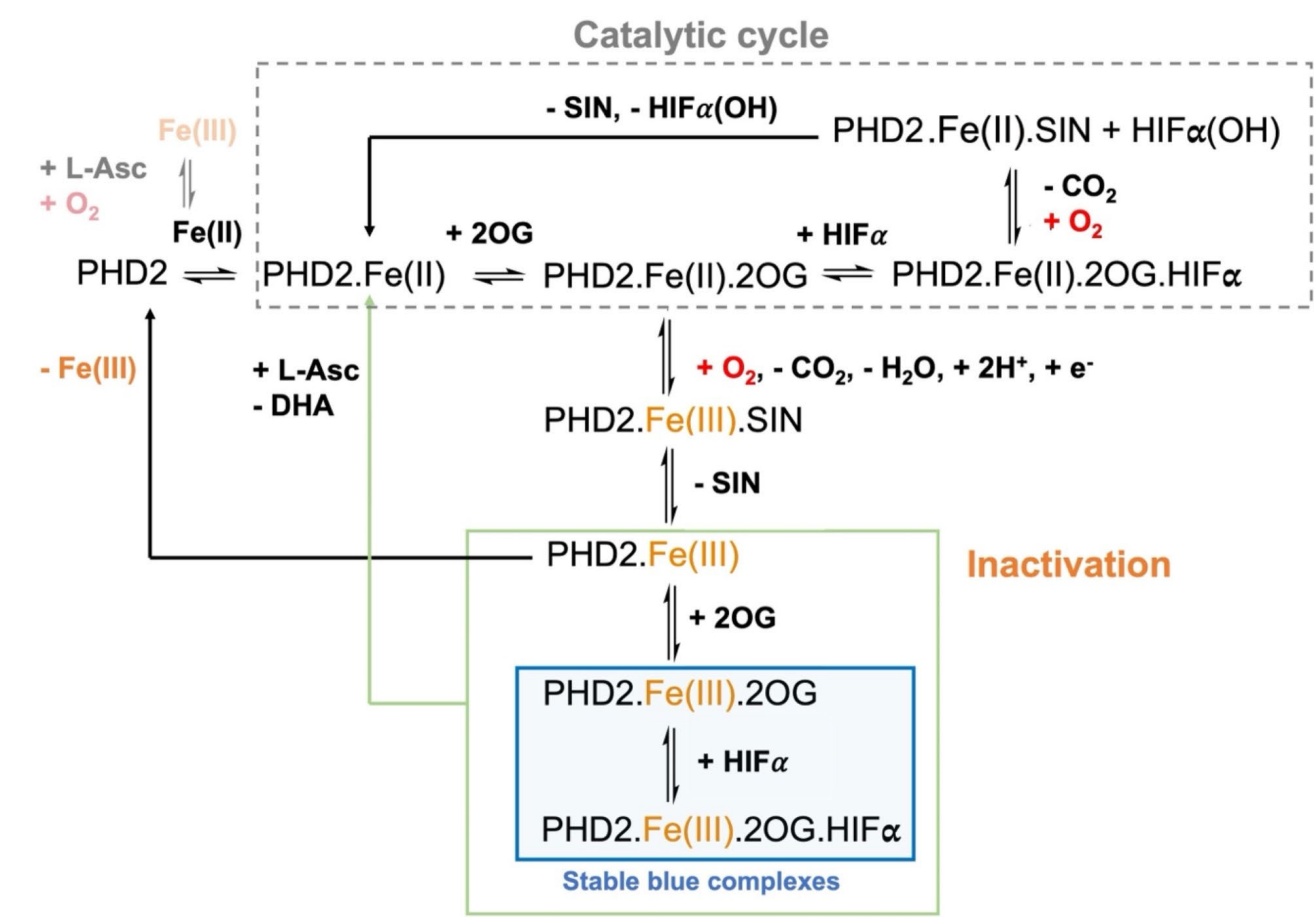
Scheme for formation of stable PHD2.Fe(III).2OG.(+/– HIFα) complexes. Note that the stoichiometries (and in some cases products) of the outlined mechanisms are uncertain. L-Asc hinders formation of the PHD2.Fe(III).2OG.(+/– HIFα) complexes and can restore them to the catalytically active PHD2.Fe(II).2OG complexes. SIN: succinate, L-Asc: L-ascorbate, DHA: dehydroascorbic acid.

The combined observations indicate that whilst uncoupled 2OG turnover may not be directly (at least, predominantly) responsible for the formation of the inactive ∼600 nm absorbance, it is likely to be responsible for the oxidation of Fe(II) to Fe(III). It is possible that Fe(III) is produced as a consequence of substrate uncoupled 2OG turnover, and the presence of L-Asc reduces this back to Fe(II), so enabling further 2OG consumption (Fig. 7). When L-Asc is not present, the PHD2.Fe(III) complex binds 2OG in preference to succinate, forming a blue chromophore (λ_max_ 596 nm).

### 2OG and 2OG derivatives have different effects on the rate of PHD2 inactivation

To further explore the role of 2OG in formation of the λ_max_ ∼600 nm absorbance, we carried out experiments replacing 2OG with methylated 2OG derivatives: 3-methyl-2OG (**1**) and 4-methyl-2OG (**2**), both of which are of biological relevance^64^ (Figs. S9b, S10b). PHD2 activity in the presence of 2OG, **1** or **2** was initially monitored under standard conditions, giving the following *K*_M_ and *k*_cat_ values: *K*_M_(2OG) = 0.09 ± 0.01 µM, *k*_cat_ (2OG) = 0.46 ± 0.07 s^− 1^, *K*_M_(**1**) = 22.6 ± 2.5 µM, *k*_cat_ (**1**) = 0.15 ± 0.01 s^− 1^_,_*K*_M_(**2**) = 3.8 ± 0.6 µM, *k*_cat_ (**2**) = 0.01 ± 0.01 s^− 1^ (Fig. S11a, b, c). As for 2OG, we monitored activity of the PHD2.Fe.2OG derivative **1** or **2** mixtures after O_2_ exposure for different times prior to incubation with HIF1α-CODD. With **1**, we observed a relatively faster decrease in activity compared with 2OG (Fig. S9a). When a PHD2.Fe(II).**1** mixture (1:0.8:10 ratio) was exposed to O_2_ for 20 min prior to HIF1α-CODD incubation, ~ 26% hydroxylation was observed, ~ 65% less than that with the O_2_-exposed PHD2.Fe.2OG mixture. When the PHD2.Fe(II).**1** mixture was kept anaerobic prior to incubation with HIF1α-CODD and O_2_, ~ 78% hydroxylation was observed (~ 52% more than that observed for the O_2_ exposed mixture) (Fig. S9a). When monitoring the UV-vis spectra of an anaerobic mixture of 300 µM PHD2, 250 µM Fe(II), and 3 mM **1** which had been exposed to O_2_, an increase in absorbance in the 350 nm region which reached a plateau after 15 min was observed, possibly due to the oxidation of Fe(II) to Fe(III) (Fig. S9b). This observation correlates with MS experiments where the maximum loss of activity was observed after 20 min.

These observations imply the rate of PHD2 activity loss in the absence of substrate can vary with the 2-oxoacid co-substrate employed. When 250 µM Fe(III) was added to 300 µM PHD2 and 3 mM **1**, the resultant spectrum showed the same feature at 350 nm as observed with the O_2_-exposed PHD2.Fe(II).**1** mixture (Fig. S9c). In accord with the MS and UV-vis data, the EPR spectrum of a mixture of PHD2.Fe(II).**1** exposed to O_2_ for 60 min showed formation of a signal corresponding to an Fe(III) species (Fig. S9d). Note, the results with 3-methyl-2OG (**1**) show it is possible for 2-oxoacids to react to form inactive PHD2.Fe(III) complexes without apparent formation of a ∼600 nm absorbance.

When the PHD2.Fe(II).**2** complex was preincubated with O_2_ for 20 min prior to incubation with HIF1α-CODD, ~ 54% hydroxylation was observed, ~ 30% more compared to the same treatment of PHD2.Fe(II).**1**, and ~ 40% less compared to the same O_2_ treatment of the PHD2.Fe(II).2OG complex (Figs. S9a, S10a). Similar to that observed with 2OG, when 3 mM **2** was added to 300 µM PHD2 and 250 µM Fe(II) and the mixture was exposed to O_2_, a feature in the 590 nm region appeared (0.009 ± 0.001 abs/min). This feature is likely representative of a PHD2.Fe(III).**2** complex (Fig. S10b, c) and the same feature was apparent when Fe(III) and **2** were added to PHD2 (Fig. S10c). The oxidation state of the ∼600 nm absorbance with **2** was shown to be (predominantly) Fe(III) by EPR, as for 2OG and **1** (Fig. S10d).

Overall, the presence of **1** or **2** causes a faster loss of PHD2 activity compared to 2OG. As with 2OG, loss of activity with **1** or **2** was clearly hindered if 1 mM L-Asc was present (Figs. S9a, S10a). Analogous experiments were performed with N-oxalylglycine (NOG), a natural catalytically inactive 2OG mimic and PHD2 inhibitor^65^. When an anaerobic mixture of 300 µM PHD2, 250 µM Fe(II), 3 mM NOG (3 mM) and 3 mM 2OG was exposed to O_2_ for 25 min, then incubated with HIF1α-CODD for 30 min prior to quenching, we observed ~ 67% hydroxylation, ~ 30% less than with only 2OG present (Fig. S12a). When monitoring the same mixture by UV-vis, we observed formation of a λ_max_ ~590 nm chromophore (Fig. S12b) with an Fe(III) oxidation state (Fig. S12c). By contrast, when an anaerobic mixture of 300 µM PHD2, 250 µM Fe(II) and 3 mM NOG was exposed to O_2_, then incubated with HIF1α-CODD and 2OG, at a ratio of 2:1 and 1:2 2OG: NOG, no activity loss was observed (Fig. S13a). By contrast with 2OG, when monitoring the UV-vis spectrum of the PHD2.Fe(II).NOG (1:0.8:10 ratio) mixture, while being exposed to O_2_, an increase in the overall absorbance was observed, but no new peak were observed to form (Fig. S13b). Similarly, we did not observe a ∼600 nm absorbance when 300 µM PHD2 was incubated with 250 µM Fe(III) and 3 mM NOG (Fig. S13c). EPR analysis of the PHD2.Fe(II).NOG, prior and after exposure of O_2_, show that the intensity of the Fe(III) signal does not increase (Fig. S13d).

Overall, these results show that whilst NOG inhibits PHD2, by competing with 2OG, when present alone it does not efficiently promote formation of PHD2.Fe(III) or PHD2.Fe(III).NOG complexes. Additionally, NOG does not, at least substantially, inhibit formation of the ∼600 nm PHD2.Fe(III).2OG chromophore when both NOG and 2OG are present in solution, under the tested assay conditions.

## Discussion

The results reveal that, in the absence of HIFα substrates, PHD2.Fe(II) reacts with 2OG and O_2_ to give an inactive PHD2.Fe(III).2OG complex which can subsequently bind HIFα substrates, as characterised by spectroscopic and crystallographic analyses. By contrast with the PHD2.Fe(II).2OG.HIF1-2α-CODD complexes, the PHD2.Fe(III).2OG complex is stable under aerobic conditions, either in the presence or absence of HIF1-2α. The PHD2.Fe(III).2OG complex manifests a peak in its UV-vis spectrum at ∼600 nm that arises on O_2_ exposure of the PHD2.Fe(II).2OG mixture, and which is observed both in the presence and absence of HIF1-2α, though on addition of HIF1-2α to the PHD2.Fe(III).2OG complex the λ_max_ is shifted. The predominant oxidation state of the Fe in the stable PHD2.Fe.2OG complex is Fe(III), as shown by EPR spectroscopy (Fig. 2). When PHD2 is mixed with Fe(III) and 2OG, a peak in the UV-vis spectrum is also observed at ∼600 nm, implying the blue colour is a consequence of the formation of a PHD2.Fe(III).2OG complex.

The solution studies are supported by crystallographic analyses. Following initial crystallographic analysis of PHD2 complexes with inhibitors^52,57^, structures of PHD2 complexed with HIF1-2α ODDs, metal and 2OG or NOG have been reported^55,56^. However, structures of catalytically active PHD2.Fe(II).2OG.HIFα complexes have not been previously described. By crystallisation under anaerobic conditions, we obtained a crystal structure of a PHD2.Fe(II).2OG.HIF2α-CODD complex (1.4 Å resolution) with an analogous PHD2.Fe(III).2OG.HIF2α-CODD complex structure being obtained under aerobic conditions (1.4 Å resolution) (Fig. 3).

The Fe(II) and Fe(III) crystal structures are strikingly similar (backbone RMSD 0.056 Å), including with respect to the presence of an Fe-bound water molecule located *trans* to the His313 and the C4 *endo*-conformation of the pyrrolidine ring of Pro531_2α − CODD_ (Figs. 3, S7). The total lack of prolyl-residue hydroxylation in the aerobic Fe(III) structures confirms the inactivity of the PHD2.Fe(III).2OG complex and is consistent with the presence of the Fe(III) oxidation state in crystals. By contrast with the PHD2.Fe(III).2OG.HIF2α-CODD crystals, the PHD2.Fe(II).2OG.HIF2α-CODD crystals were active - exposure of them to O_2_ results in PHD2 in complex with C4 hydroxylated-Pro531_HIF2α − CODD_ (PHD2.HIF2α-CODD(OH)). The anaerobic crystallisation method described for the PHD2.Fe(II).2OG.HIF1-2α-CODD complex using a crystal form wherein the HIF2α-CODD(OH) product is trapped could be used for future time-resolved crystallography studies concerning the mechanism of PHD2, as has been done for isopenicillin N synthase, which is structurally related to the OGO^30^.

The overall folds of the PHD2.Fe(II).2OG.HIF2α-CODD and PHD2.HIF2α-CODD(OH) product structures are similar (RMSD 0.071 Å). In the PHD2.HIF2αCODD(OH) structure, no density corresponding to the active site Fe or 2OG/succinate was observed, though hydroxylated Pro531_HIF2α − CODD_ was clearly present (Fig. 3). The lack of Fe, 2OG and succinate, but the presence of the HIF2α-CODD(OH) product, is likely predominantly due to crystal lattice effects that disrupt the normal solution order of product and succinate release, but which promotes product trapping. There is, however, a possibility that apo-forms of the PHDs may bind HIFα proteins within cells, especially when Fe or 2OG concentrations are limiting.

In the HIF2α-CODD(OH) product complex structure, the pyrrolidine ring of Pro531_HIF2α − CODD_ appears to adopt the C4-*endo* conformation (Fig. 3). In crystal structures of pVHL.elonginB/C complexed with prolyl hydroxylated HIFα, prolyl residues are observed in the C4-*exo* conformation (PDB: **1LQB**, **6I7R**)^59,60^, a state stereoelectronically favoured by *trans*-4-prolyl hydroxylation^61^. Although further validation is required, our results suggest the proposed key role of conformational changes of hydroxylated proline residues in the hypoxic response^61^ may occur after release of the hydroxylated product from PHD2. Further, the active site presence of the HIF2α-CODD(OH) product, but not substrate, may promote release of succinate and hinder 2OG binding; indeed, there is evidence that in solution the active site presence of hydroxylated product hinders binding of 2OG to the PHD2.Fe(II) complex^66^.

The mechanism of formation of the PHD2.Fe(III).2OG complex likely involves initial (near) stoichiometric turnover to give succinate and CO_2_, as shown by NMR analysis. This initial reaction can give an oxidised form of the active site Fe, potentially Fe(III), which can bind with excess 2OG in solution. When 2OG is replaced with catalytically inert NOG, PHD2.Fe(II) was not observed to react giving PHD2.Fe(III) or PHD2.Fe(III).NOG complexes (Fig. S13). These observations support the importance of the initial HIFα substrate-uncoupled turnover of 2OG or catalytically active 2OG analogues to give a PHD2.Fe(III) complex, which can then bind 2OG. The PHD2.Fe(III).2OG complex can bind HIFα to give an inert PHD2.Fe(III).2OG.HIFα complex. Precisely how Fe(II) is converted to Fe(III) at the active site during uncoupled turnover is unclear, but may involve reaction of the PHD2.Fe(II).2OG complex with O_2_ to give CO_2_ and PHD2.Fe(IV).succinate complex which is reduced by a one electron process (Fig. 7).

It is unclear how L-Asc functions in 2OG oxygenase catalysis, especially *in vivo*. One possibility is that L-Asc enables direct or indirect reduction of catalytically inert Fe(III) or Fe(IV) species (formed either in solution or at the active site) to restore catalytically active Fe(II)^42,67,68^. In some OGO, L-Asc consumption is stoichiometrically coupled with substrate uncoupled turnover of 2OG, again helping to maintain a catalytically active form^40^. In accord with our results it is also possible that L-Asc reduces Fe(IV) or Fe(III) species forming during uncoupled turnover of 2OG to succinate, thus promoting optimal activity^42,68^.

Our results show L-Asc, and to a lesser extent IAA, hinders formation of the PHD2.Fe(III).2OG complexes and, hence, PHD2.Fe(III).2OG.HIFα complexes. L-Asc and IAA slowly cause regeneration of the catalytically PHD2.Fe(II) complex from the PHD2.Fe(III).2OG complex. Interestingly, neither of the thiol reducing agents tested, that is the biologically important reducing peptide glutathione and DTT (a reducing agent commonly used in OGO assays buffers^42^), hindered formation of the λ_max_ ~600 nm chromophore or reactivated it, suggesting a potentially selective role for L-Asc type reducing agents.

The precise mechanisms underlying our L-Asc mediated observations are unclear, and can be the subject of further investigations, which are challenging due to the complex nature of the redox chemistry involved, not only including the ability of L-Asc to reduce Fe(III), but its capacity to react with O_2_ to generate hydrogen peroxide, a reaction inhibited by Fe(II)^63,69^.

The situation the potential to be even more complex in cells, where multiple exogenous and endogenous redox agents are present, including electron rich compounds related to L-Asc such as catechols. The results highlight that care should be taken in optimising the precise conditions for OGO assays. In particular, they imply that incubation of OGO with Fe(II), 2OG, and O_2_, prior to substrate addition, might lead to formation of an inactive Fe(III) complex, at least in the absence of sufficient amount of an appropriate reducing agent. The results should also help guide optimised conditions for use of isolated OGO in preparative biocatalysis, including with respect to the regeneration of inactivated OGO.

Given the role of certain reducing agents (L-Asc/IAA) in hindering formation of the inactive PHD2.Fe(III) complexes, the presence or not, and timing of addition of reducing agents (including L-Asc) may account for some reported differences in OGO assay results, including in kinetic and inhibitor parameters. It seems, however, unlikely that this can explain differences in results concerning non-HIFα PHD substrates^21^. Nonetheless, it is possible that the formation of catalytically inactive PHD.Fe(III).2OG complexes in cells may enable capture of substrate and, maybe, non-substrate proteins at the PHD (and other OGO) active sites (see below). Note that both catalytically productive and non-productive interactions will likely be disrupted by OGO Fe-ligand mutagenesis (and some inhibitors^70^), a common control used in cell experiments.

The presence of OGO.Fe(III) complexes (+/-2OG) may also explain why, at least in part, it is possible to sometimes capture substrates in immunoprecipitation/related substrate ‘fishing’ experiments with OGO, in the absence of inhibitors such as NOG^65,70^. Further controls that may be considered in cellular substrate investigation studies might involve analyses with and without L-Asc or comparing potential substrate capture results with and without NOG/other inhibitors, though it is appreciated these introduce another variable to already technically complex and expensive studies. It also cannot be ruled out that some PHD/other OGO Fe(III) complexes have as yet undiscovered catalytic and/or stoichiometric reactivities.

Although, it seems unlikely that our observations with PHD2 will be universally applicable to OGO, it is of interest to investigate whether they apply to other OGO. In this regard it will also be of interest to screen 2-oxo acids other than 2OG; the results presented here with biologically relevant methylated 2OG derivatives (**1**,**2**)^64^, reveal potential for different kinetics and different outcomes with respect to inactivation with different 2-oxoacid co-substrates (Figs. S9, S10). Notably formation of a λ_max_ ~600 nm absorbance was observed with 2OG and 4-methyl-2OG (**2**), but not with 3-methyl-2OG (**1**) (Figs. S9, S10). The reason for the difference in reactivity of 2OG, **2** and **1** is unclear, but may reflect the precise coordination mode / keto-enol tautomeric state of the 2-oxoacid group which is likely more influenced by the C3-methylation (as in **2**) as compared to the C4-methylation (as in **1**).

A blue colour has been observed with purified forms of other OGO, including, taurine dioxygenase (TauD^53^), TfdA^54^ and FIH^71^, and an inactive state of 4-hydroxyphenylpyruvate dioxygenase (HPPD), a non-heme Fe-oxygenase which does not belong to the OGO structural family^72^. HPPD^22^ can be purified in a blue Fe(III) form, which, like PHD2, can be reactivated by addition of L-Asc^72^. The auto-hydroxylation of active site proximate residues has been suggested as a possible explanation for formation of the blue chromophore. For HPPD, this proposal remains to be validated^73,74^, though self-hydroxylation has been reported for other OGO including JMJD6 and AlkB homologs^41,51,71,75–77^. Despite careful analyses (including of the structures reported here), we did not observe any evidence for self-hydroxylation either in solution or crystallographic studies on PHD2, for which the appearance of a blue colour results from binding of 2OG to the PHD2.Fe(III) to give a PHD2.Fe(III).2OG complex.

Most reported OGO inhibitors, including drugs targeting the PHDs, ligate to the active site Fe(II), as observed by spectroscopic and crystallographic analysis^52,70^, with the γ-butyrobetaine hydroxylase inhibitor mildronate being an exception^78^. Given the stability of the PHD2.Fe(III).2OG complexes, it will be of interest to test whether known OGO inhibitor classes, including oncometabolites such as (2*R*)-hydroxyglutarate^79–81^ bind to, or even react with, OGO.Fe(III) complexes. It is also of interest to identify mechanism-based inhibitors that actively promote formation of OGO.Fe(III) complexes in cells. In this regard studies showing some 2-oxoacid 2OG analogues, including some present in the human diet^64,82,83^ can act as 2OG replacements or inhibitors, depending on the OGO are of interest^64,84,64^.

As with all biochemical studies, the relevance of our results to biology needs to be investigated. The absence of methods to measure the Fe redox state at a specific enzyme active site in cells makes this challenging. In this regard it is of interest, both from research and pharmaceutical applications, to develop inhibitors that are selective for the Fe(II) or the Fe(III) forms of the PHDs and other OGO.

Several factors indicate our results are worthy of functional consideration in terms of the roles of the PHDs in the hypoxic response. Although ‘targeting’ processes may affect local concentrations of individual components, it would seem likely that the intracellular concentrations of Fe(II)/Fe(III), 2OG, and O_2_ (in normoxic conditions) are in excess relative to the concentrations of the PHDs and the HIFα isoforms, the latter of which are efficiently degraded^1,3,86^. It is thus possible that, in the absence of other factors, in cells the PHDs are exposed to sufficient quantities of Fe(II), 2OG and O_2_ that enable formation of stable PHD.Fe(III).2OG complexes, which may help them capture HIFα. Note, there is extensive evidence that OGO, including PHD2, bind their substrates more efficiently if 2OG is already bound at the active site^33–37,66^. Another possibility is that the PHD2.Fe(III).2OG.(+/- HIFα) complexes are involved in transport prior to Fe(III) reduction and subsequent encounter with O_2_. As noted above, these proposals require a reducing agent to activate the PHD2.Fe(III).2OG complexes for catalysis. It is possible that L-Asc acts in this manner, though the rate of activation mediated by it was slow under our conditions, hence identifying biologically relevant activation processes is important.

The formation of PHD2.Fe(III).2OG.HIFα complexes is also of potential interest in the context of kidney cancer where mutations to VHL (VHL1) gene lead to HIFα upregulation and predisposition to clear cell renal cell carcinoma (ccRCC)^87^. Therapeutic promotion of the formation and degradation of PHD2.Fe(III).2OG.HIF2α complexes using small-molecules may be a complementary strategy for treatment of VHL mutant associated renal cell carcinoma (Fig. S15).

## Experimental procedures

### General methods and materials

Reagents were from Merck Life Sciences UK Ltd., Apollo Scientific, or Fisher Scientific, unless otherwise stated. 2OG derivatives **1** and **2** were prepared as reported^84^. HIF1α_556−574_-CODD (DLDLEMLAPYIPMDDDFQL) and HIF2α_523−542_-CODD (ELDLETLAPYIPMDGEDFQL) (prepared with C-terminal amides) were from GL Biochem Ltd. Stock solutions of co-substrate/cofactor ((+)-sodium L-ascorbate, L-Asc; 2-oxoglutarate, 2OG; ammonium iron(II) sulfate hexahydrate, FAS, (NH_4_)_2_Fe(SO_4_)_2_·6H_2_O) and assay buffers were freshly prepared. Anaerobic handling of samples was performed in a Belle Technologies anaerobic chamber filled with nitrogen (O_2_ < 3 ppm). Recombinant PHD2_181 − 407_ was produced and purified as described in Supplementary Methods.

### Spectroscopy

UV-vis spectra were collected at 20 °C using an Agilent Cary 3500 UV-vis Compact Peltier spectrometer. For anaerobic sample preparation, the reagents were equilibrated in an anaerobic chamber for 20 min prior to analysis. Samples were transferred into a quartz cuvette sealed with a Suba-Seal^®^ (Sigma Aldrich) while performing the UV-vis data collection to maintain anoxic conditions.

Continuous-wave EPR spectra of PHD2_181 − 407_ samples were obtained at a temperature of 10 K and a frequency of 9.3864(4) GHz with a modulation amplitude of 1 mT and a microwave power of 100 µW using a Bruker BioSpin EMXmicro Premium bridge with a Bruker ER4122-SHQE-W1 resonator and Oxford Instruments ESR900 helium flow cryostat controlled by an Oxford Instruments ITC-503 S temperature controller.

EPR Spectroscopy was performed at X-band in the Centre for Advanced ESR (CAESR) of the Department of Chemistry, University of Oxford.

### Crystallography

Crystals were obtained using the sitting-drop method. PHD2_181 − 407_.Fe(III).2OG.HIF2α-CODD_523 − 542_ crystals were obtained aerobically. The PHD2_181 − 407_.Fe(II).2OG.HIF2α-CODD_523 − 542_ crystals were obtained anaerobically. PHD2_181 − 407_.product crystals were obtained by exposure of PHD2_181 − 407_.Fe(II).2OG.HIF2α-CODD_523 − 542_ crystals to O_2_. See Supplementary Methods for details.

### Assays

Solid-Phase extraction-MS activity assays, including activity assays for PHD2.Fe(III).2OG complexes and ^1^H NMR assays employed reported procedures or variations thereof, and are described in Supplementary Methods.

## Electronic supplementary material

Below is the link to the electronic supplementary material.

Supplementary Material 1

## Data Availability

Coordinates and structure factors for all reported complex structures are deposited in the Protein Data Bank. The PDB ID codes are: **8Q5S** for PHD2_181 − 407_.ACT.HIF2α-CODD_523 − 542_, **8Q6D** for PHD2_181 − 407_.Fe(II).2OG.HIF2α-CODD_523 − 542_, **8Q6E** for PHD2_181 − 407_.Fe(III).2OG.HIF2α-CODD_523 − 542_ and **8Q64** for PHD2_181 − 407_.ACT.HIF2α-CODD_523 − 542_(OH). Other data will be made available upon request to Christopher J. Schofield (christopher.schofield@chem.ox.ac.uk).
