# Supplementary material for: Human prolyl hydroxylase domain 2 reacts with O2 and 2-oxoglutarate to enable formation of inactive Fe(III).2OG.hypoxia-inducible-factor α complexes
Source: Sci Rep. 2024 Oct 30;14:26162. doi: 10.1038/s41598-024-75761-y (PMC11525979; doi:10.1038/s41598-024-75761-y)
Supplement: Supplementary file 1 — Supplementary Material 1 [file 41598_2024_75761_MOESM1_ESM.docx]

***Supporting information***

**Human prolyl hydroxylase domain 2 (PHD2) reacts with O_2_ and 2-oxoglutarate (2OG) to enable formation of inactive Fe(III).2OG.Hypoxia-Inducible-Factor (HIFα) complexes**

Giorgia Fiorini^1^, Stephen A. Marshall^1^, William Jr Figg^1^, William K. Myers^2^, Lennart Brewitz^1^, and Christopher J. Schofield^1^

^1^Chemistry Research Laboratory, Department of Chemistry and the Ineos Oxford Institute for Antimicrobial Research, 12 Mansfield Road, Department of Chemistry, University of Oxford, Oxford, OX1 3TA, United Kingdom.

^2^ Inorganic Chemistry Laboratory, Department of Chemistry, South Parks Road, Oxford, OX1 3QR, United Kingdom.

**Table of Contents**

Figure S1. Proposed mechanisms for 2OG oxygenases adapted for PHD2 and FIH. 3

Figure S2. UV-vis spectra providing evidence for formation of a stable PHD2.Fe(III).2OG.HIF1α-CODD complex. 4

Figure S3. Temperature dependence of the blue chromophore generated by exposing PHD2.Fe(II).2OG to O_2_ 4

Figure S4. UV-vis spectra of an anaerobic and O2 exposed mixture of PHD2, Fe(III) +/- 2OG providing evidence that the PHD2.Fe(III).2OG complex is blue **(λmax 598 nm)** and view of the blue chromophore in solution. 5

Figure S5. Deconvoluted electrospray ionisation LC-MS spectra of purified PHD2 and PHD2.Fe(II).2OG prior and after O_2_ exposure showing a lack of evidence for PHD2 self-hydroxylation. 5

Figure S6. Comparison of the PHD2_181-407_.Fe(II)/Fe(III).2OG.HIF2α-CODD_523-542_ with the PHD2_181-407_.Fe(II).2OG.HIF2α-CODD_523-542_ and PHD2_181-407_.ACT.HIF2α-CODD(OH)_523-542_ crystal structures. 6

Figure S7. Views of the active site residues and Pro531/Hyp531_2αCODD_ conformations in the PHD2_181-407_.Fe(II)/Fe(III).2OG.HIF2α-CODD_523-542_/HIF2α-CODD(OH)_523-542_ complex crystal structures. 7

Figure S8. 1H NMR (700 MHz) time course measurements showing the effect on L-ascorbate on HIFα substrate ‘uncoupled’ 2-oxoglutarate turnover in the presence and absence of PHD2_181-407_ 8

Figure S9. Hydroxylation assays and UV-vis spectra displaying the effect of O_2_ exposure on the PHD2.Fe(II).[1] complex. 9

Figure S10. Hydroxylation assays and UV-vis spectra investigating the effects of O_2_ exposure on the PHD2.Fe(II).[2] complex. 10

Figure S11. Steady-state kinetics at different concentration of 3-methyl-2OG, 2OG and 4-methyl-2OG (n=3). 11

Figure S12. Hydroxylation assays and UV-vis spectra investigating the effect of O_2_ exposure on the mixture containing PHD2, Fe(II), NOG and 2OG. 12

Figure S13. Hydroxylation assays and UV-vis spectra investigating the effect of O_2_ exposure on the PHD2.Fe(II).NOG complex. 13

Figure S14. Rates of formation of the PHD2.Fe(III).2OG complex in the presence of different reducing agents. 14

Figure S15. Summary of the implications, questions arising, potential applications and roles linked to the observation of stable PHD2.Fe(III).2OG.(+/- HIFα) complexes. 14

Table S1. Data collection and refinement statistics for the PHD2_181-407_ crystal structures 15

Supplementary Methods 16

Protein production and purification 16

Solid-phase extraction-MS activity assays 16

^1^H NMR assays 18

Crystallography 19

Crystallographic data processing 19


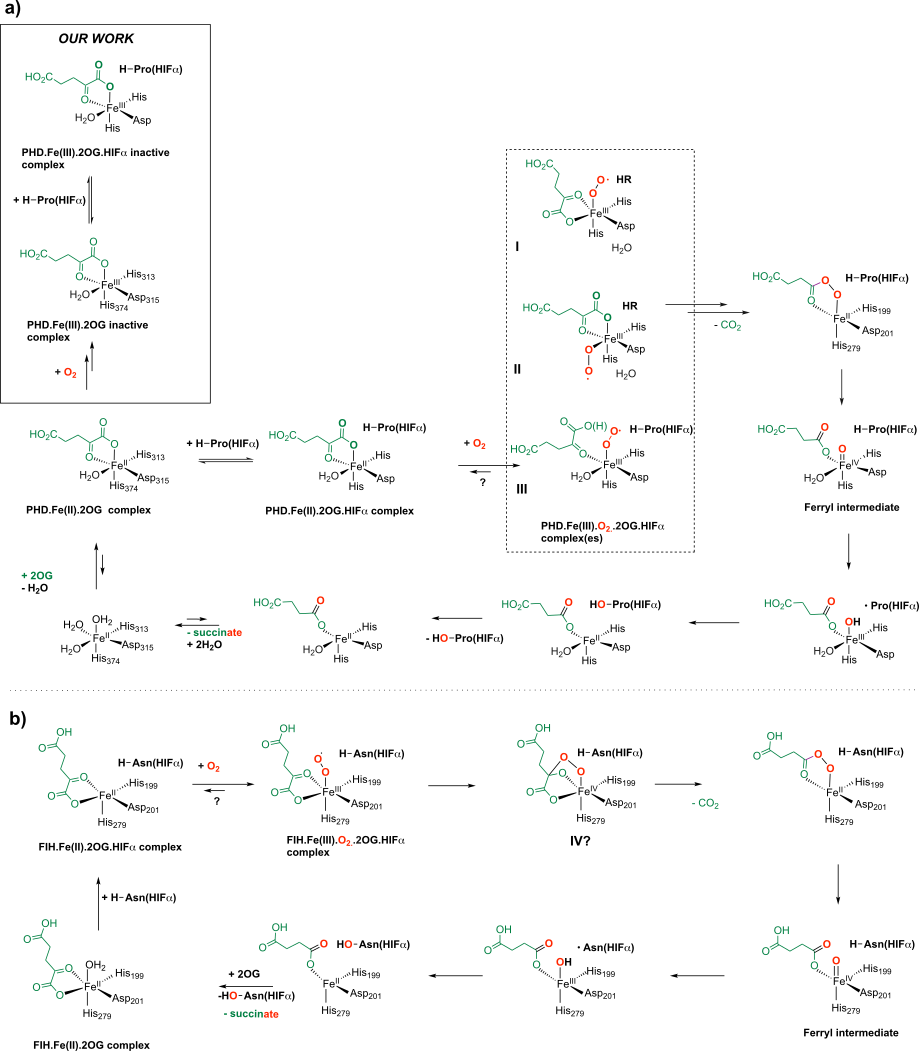


Figure S1. Proposed mechanisms for 2OG oxygenases adapted for PHD2 and FIH. The schemes show modifications of the current consensus overall mechanism for 2OG dependent oxygenases. The box summarises a key new observation reported here. (a) In the PHD2 resting state, the active site Fe(II) is coordinated by two histidine residues, an aspartate residue, and three water molecules. 2OG binds to the Fe(II) as a bidentate ligand with displacement of two water molecules. The 2OG C1 carboxylate is observed by crystallography to coordinate *trans* to His374^1^; the substrate proline-residue binds proximal to the Fe(II). The precise mode of O_2_ binding/superoxide formation for PHD2 is uncertain; possibilities are shown (intermediates I, II and III)^2^. 2OG decarboxylation may occur through a cyclic intermediate (shown as IV for FIH in (b))^3,4^ to give a succinyl peroxide intermediate (which has been observed for another 2OG oxygenase^5^), leading to the formation of an Fe(IV)=O species responsible of substrate hydroxylation. (b) The FIH.Fe(II).2OG.substrate complex typically presents a vacant coordination site^6^ where O_2_ is proposed to bind to give a Fe(III)-superoxo intermediate that can rearrange to give a Fe(IV)=O species possibly via a cyclic intermediate. As with the PHDs, a Fe(IV)=O species is likely responsible for substrate hydroxylation^7^.

**
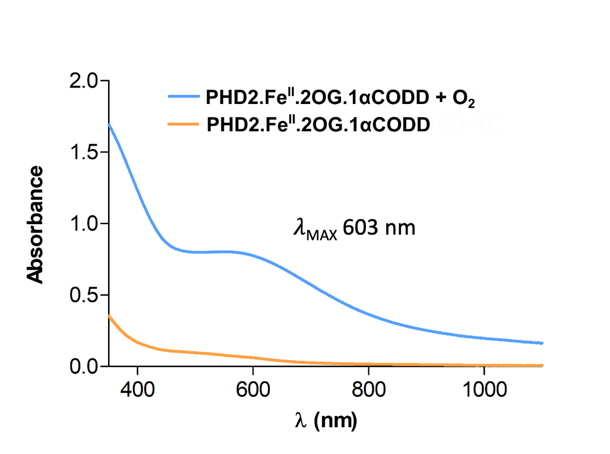
**

Figure S2. UV-vis spectra providing evidence for formation of a stable PHD2.Fe(III).2OG.HIF1α-CODD complex. UV-vis spectra of an O_2_ exposed (blue colour) and anaerobic (orange colour) mixture of PHD2_181-407_ (300 μM), (NH_4_)_2_Fe(II)(SO_4_) (250 μM), 2OG (3 mM) and HIF1α-CODD_556-574_ (250 μM).

**
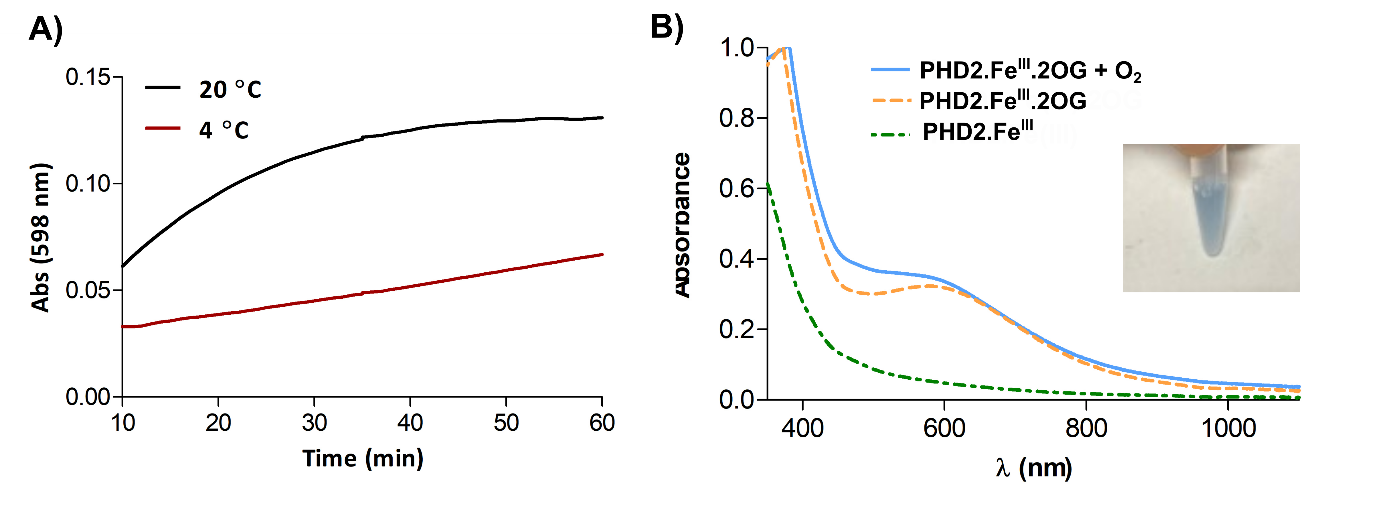
**

Figure S3. Temperature dependence of the blue chromophore generated by exposing PHD2.Fe(II).2OG to O_2_ (A) Formation of the blue chromophore (λ_max_ 598 nm) is faster at 20 °C than at 4 °C. A solution containing 300 µM PHD2_181-407_, 250 µM Fe(II) and 3 mM 2OG (in 50 mM MOPS buffer) was exposed to O_2_, at 20°C or 4°C in a Peltier controlled cuvette holder.

**
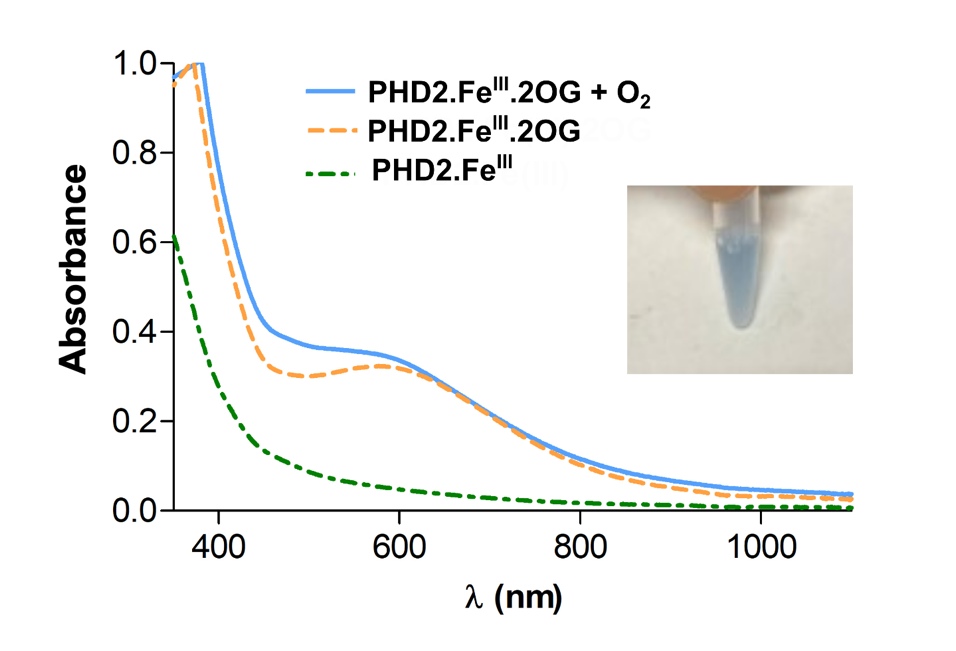
**

Figure S4. UV-vis spectra of an anaerobic and O2 exposed mixture of PHD2, Fe(III) +/- 2OG providing evidence that the PHD2.Fe(III).2OG complex is blue (λmax 598 nm) and view of the blue chromophore in solution. PHD2_181-407_ (300 μM), Fe_2_(SO_4_)_3_ (250 μM), 2OG (3 mM when present).

**
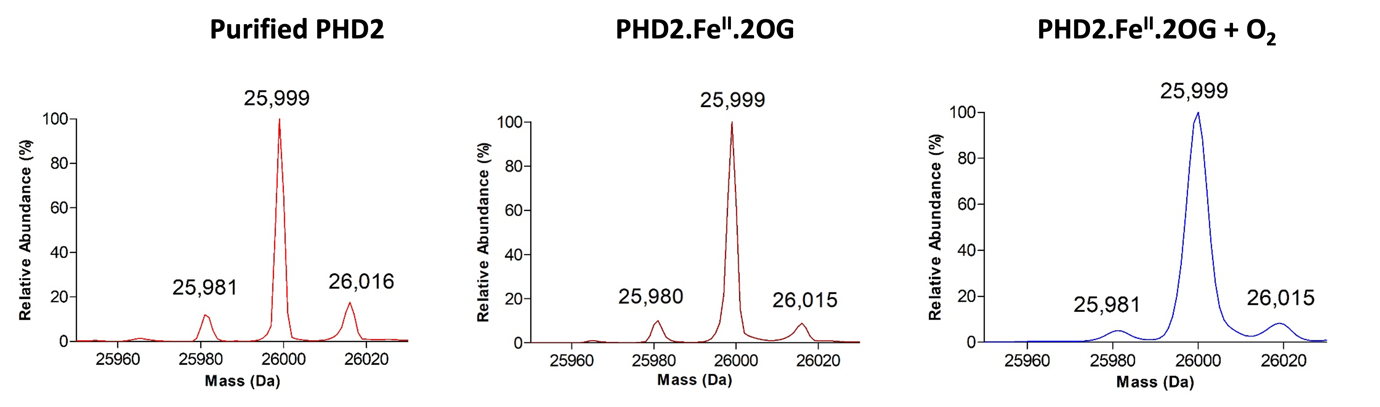
**

Figure S5. Deconvoluted electrospray ionisation LC-MS spectra of purified PHD2 and PHD2.Fe(II).2OG prior and after O_2_ exposure showing a lack of evidence for PHD2 self-hydroxylation. Data were collected as described in the Supplementary Methods. Calculated mass for PHD2= 25,999 Da, as observed.

**
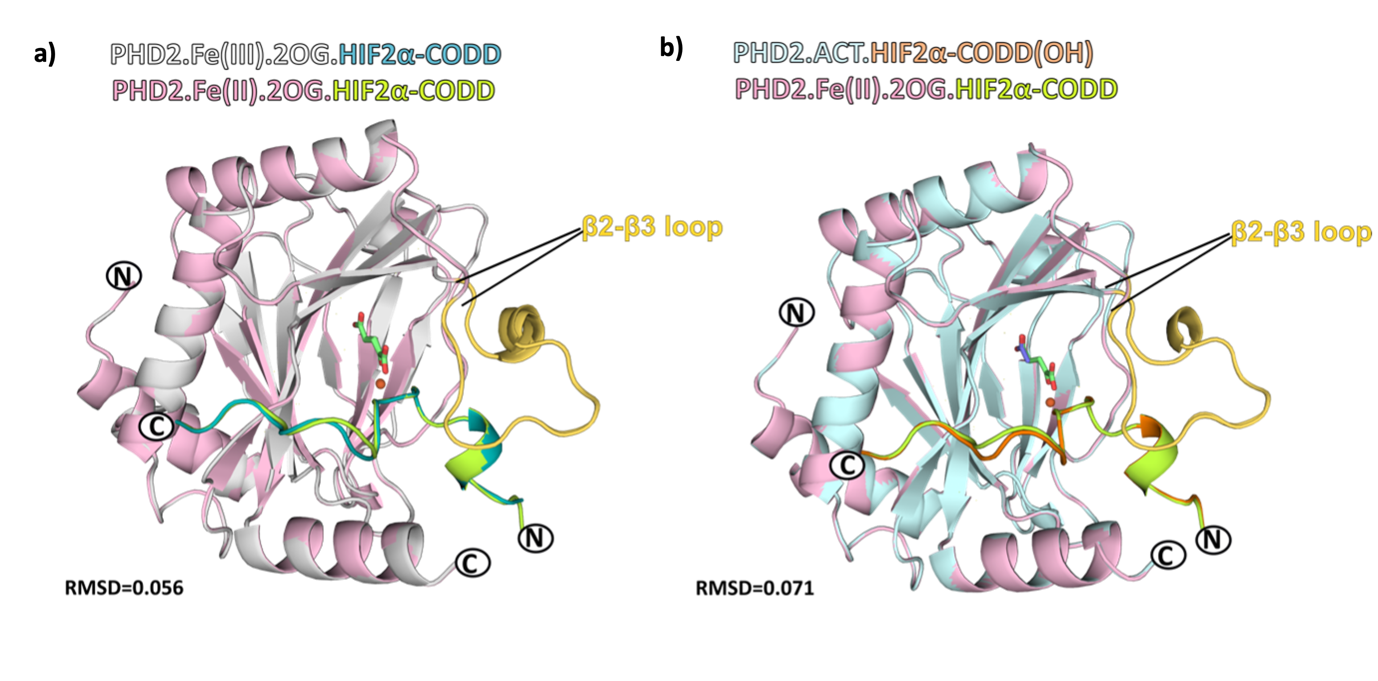
**

Figure S6. Comparison of the PHD2_181-407_.Fe(II)/Fe(III).2OG.HIF2α-CODD_523-542_ with the PHD2_181-407_.Fe(II).2OG.HIF2α-CODD_523-542_ and PHD2_181-407_.ACT.HIF2α-CODD(OH)_523-542_ crystal structures. (a) Structural superimposition of the PHD2_181-407_.Fe(II).2OG.HIF2α-CODD_523-542_ complex structure (PDB: 8Q6D) with the PHD2_181-407_.Fe(III).2OG.HIF2α-CODD_523-542_ complex structure (PDB: 8Q6E) showing the two structures are strikingly similar (RMSD 0.056 Å). (b) Structural alignment of PHD2_181-407_.Fe(II).2OG.HIF2α-CODD_523-542_ complex structure (PDB: 8Q6D) with the PHD2_181-407_.ACT.HIF2α-CODD(OH)_523-542_ complex structure (PDB: 8Q64) showing high overall structural similarity (RMSD 0.071 Å). Cα RMSD values were calculated using the PyMOL align command. ACT: acetate ion.


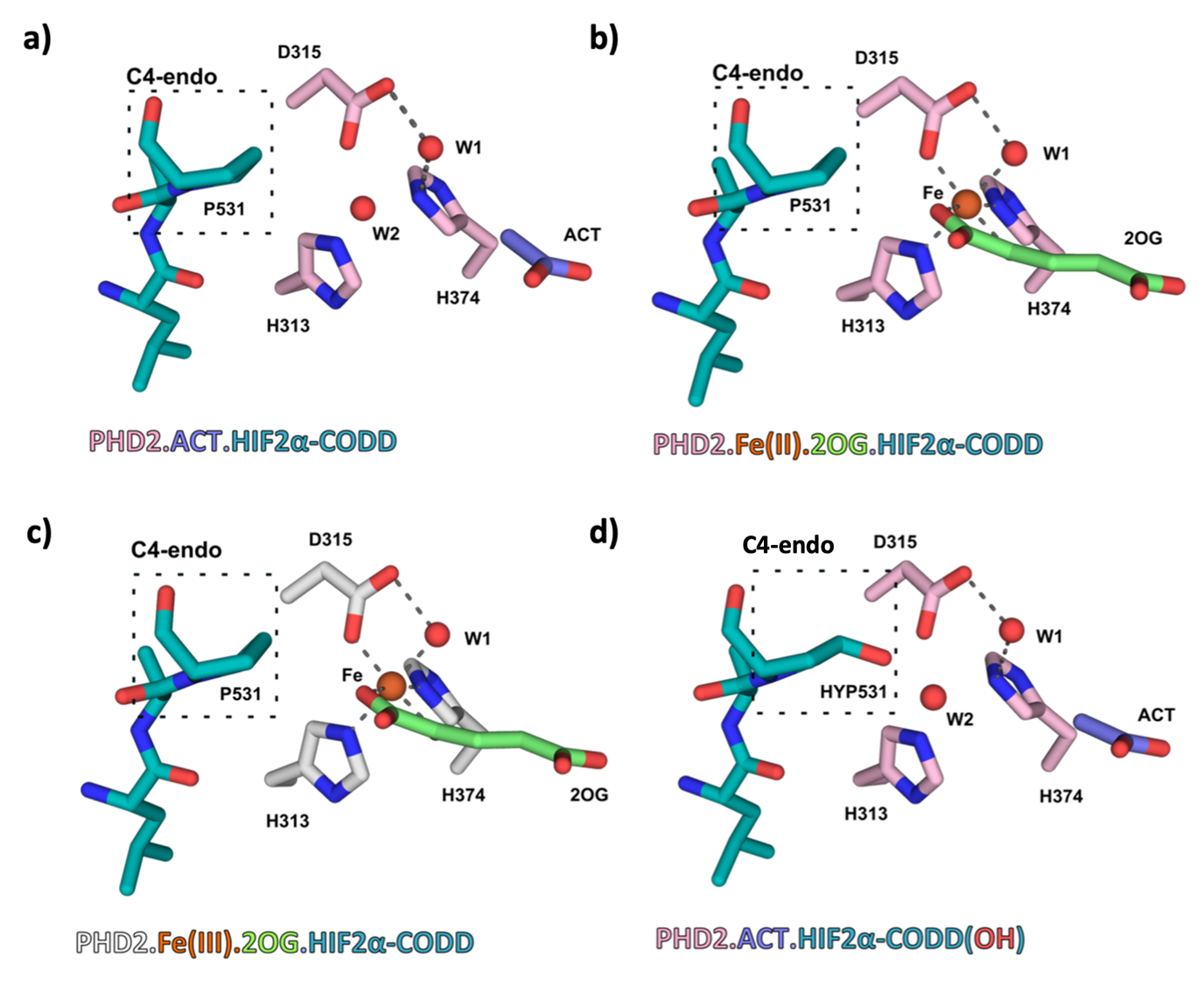


Figure S7. Views of the active site residues and Pro531/Hyp531_2αCODD_ conformations in the PHD2_181-407_.Fe(II)/Fe(III).2OG.HIF2α-CODD_523-542_/HIF2α-CODD(OH)_523-542_ complex crystal structures. (a) View of the acetate ion (ACT) (purple), water and the LAPY residues from HIF2α-CODD_523-542_ in the PHD2_181-407_.ACT.HIF2α-CODD_523-542_ complex structure (PDB: 8Q5S). (b) View of 2OG (green sticks), Fe, water and LAPY residues from HIF2α-CODD_523-542_ (white sticks) in the PHD2_181-407_.Fe(II).2OG.HIF2α-CODD_523-542_ complex structure (PDB: 8Q6D). (c) View of 2OG (green sticks), Fe, water and LAPY residues from HIF2α-CODD_523-542_ in the PHD2_181-407_.Fe(III).2OG.HIF2α-CODD_523-542_ complex structure (PDB: 8Q6E). (d) View of ACT (purple), water and LAPY residues from hydroxylated HIF2α-CODD_523-542_ in the PHD2_181-407_.ACT.HIF2α-CODD(OH)_523-542_ complex structure (PDB: 8Q64). Polar interactions: black dashes; waters: red), Fe: (orange spheres); LAP CODD_523-542_ residues: blue sticks.

**
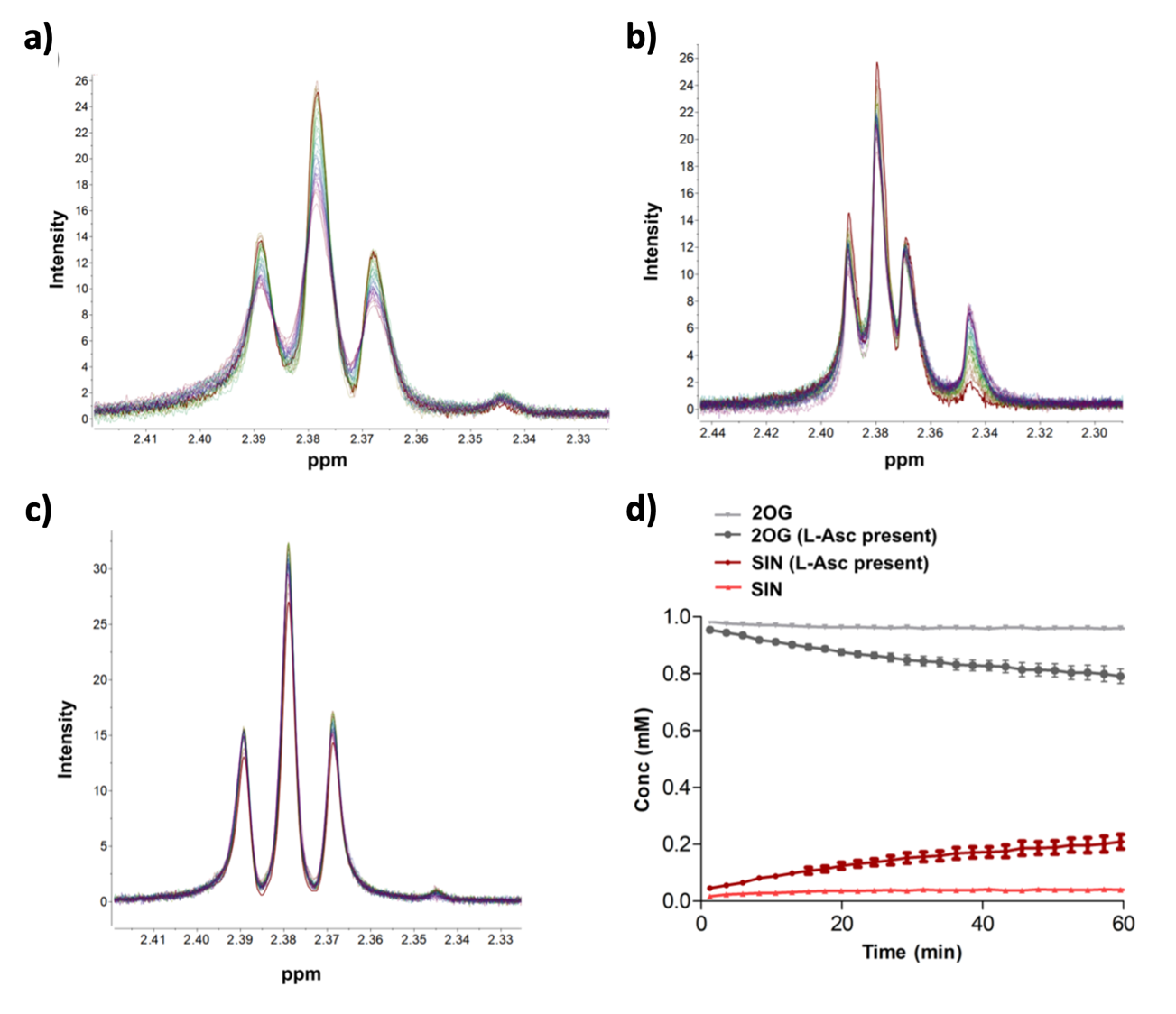
**

Figure S8. 1H NMR (700 MHz) time course measurements showing the effect on L-ascorbate on HIFα substrate uncoupled 2-oxoglutarate turnover in the presence and absence of PHD2_181-407_ (n=3). (a) A mixture of 20 µM PHD2_181-407_ was added to 50 µM (NH_4_)_2_Fe(II)(SO_4_) and 1 mM 2OG. The 2OG triplet at 2.38 ppm was monitored over 60 minutes. Consistent with a prior report ^8^ only a low level (~3 %, 30 µM) of 2OG was converted to succinate (singlet at 2.33 ppm). (b) A mixture of 20 µM PHD2_191-407_ was added with 50 µM Fe(II), 4 mM L-Asc and 1 mM 2OG. The 2OG triplet (corresponding to the hydrogen atoms on C4) at 2.38 ppm was monitored over 60 minutes; an increase in the succinate singlet (2.33 ppm) can be clearly observed when compared with the spectrum obtained without L-Asc. The amount of 2OG converted to succinate (singlet at 2.33 ppm) was ~15%. (c) A mixture of 50 µM (NH_4_)_2_Fe(II)(SO_4_), 1 mM 2OG and 4 mM L-Asc was O_2_ exposed and monitored (60 minutes). Note that the succinate singlet (2.33 ppm) does not increase substantially over time (~1% of 2OG is converted to succinate over 60 minutes in the absence of PHD2). (d) Concentrations of 2OG and succinate, obtained from the integration of the ^1^H NMR signals (See Supplementary Methods for details), plotted over time. Results are means of 3 independent replicates (n= 3, mean ± SD).


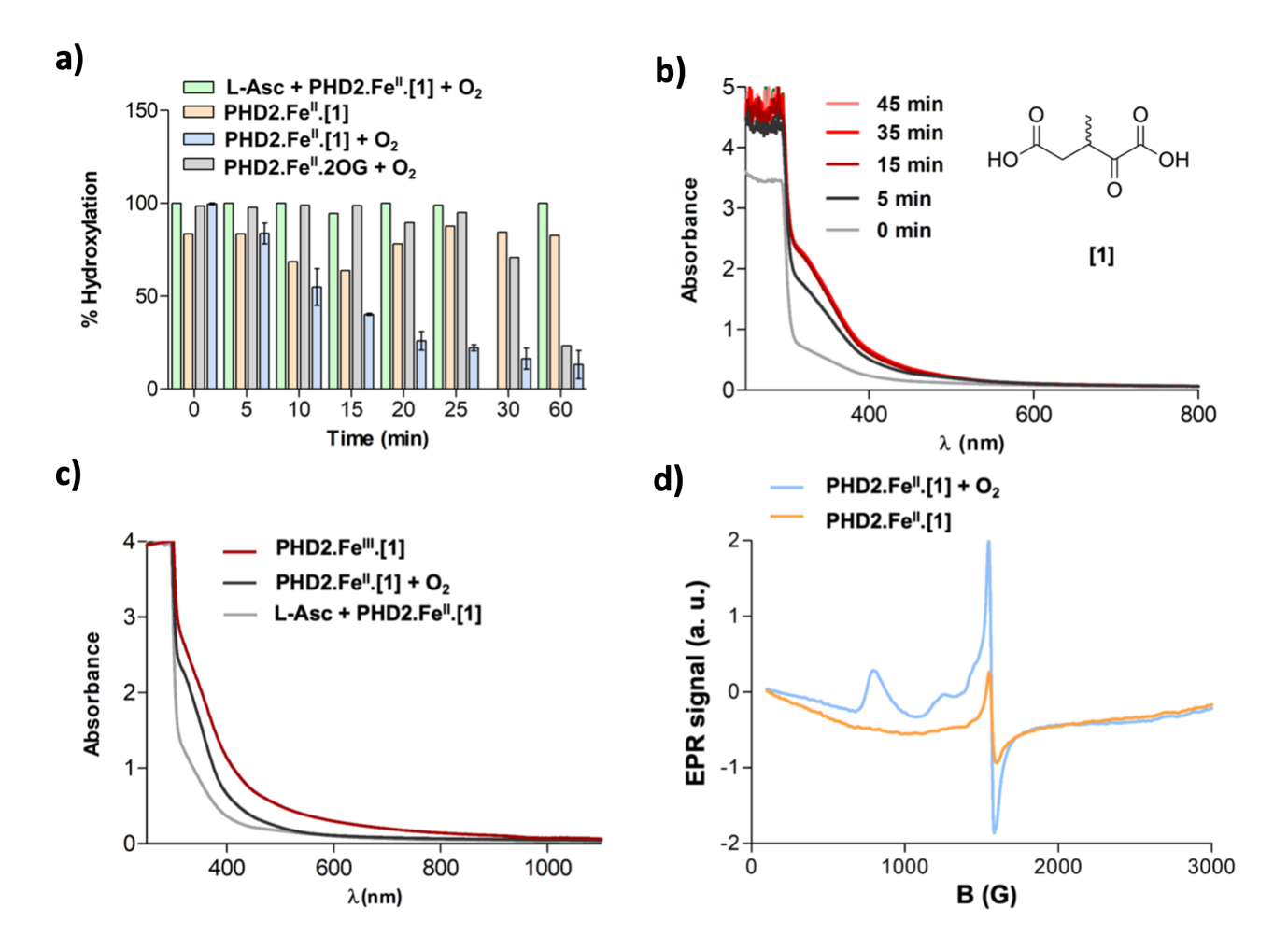


Figure S9. Hydroxylation assays and UV-vis spectra displaying the effect of O_2_ exposure on the PHD2.Fe(II).[1] complex. (a) The extent of hydroxylation when a PHD2.Fe(II).[1] mixture is added to a HIF1α-CODD_556-574_ solution, with and without prior O_2_ exposure of the PHD2.Fe(II).[1] mixture (blue and orange bars), and with L-Asc added to the a PHD2.Fe(II).[1] mixture prior to O_2_ exposure (green bars). Grey bars: The extent of hydroxylation when a PHD2.Fe(II).2OG mixture is added to an HIF1α-CODD_556-574_ solution, with prior O_2_ exposure of the PHD2.Fe(II).2OG mixture. The results are means of 3 independent repeats (n=3, mean ± SD). (b) Time-course of the UV-vis spectra of 300 µM PHD2, 250 µM (NH_4_)_2_Fe(II)(SO_4_), 3 mM [1] following exposure to O_2_. (c) Comparison of the UV-vis spectra of PHD2.Fe(II).[1] (black), PHD2.Fe(III).[1] (red) and PHD2.Fe(II).[1] in the presence of 1 mM L-Asc (grey). (d) EPR spectra of anaerobic PHD2.Fe(II).[1] (orange) and O_2_ exposed (60 minutes) PHD2.Fe(II).[1] (blue). The % hydroxylation is calculated as described in the Supplementary Methods and was obtained by extracting the data for the +2 charge states of the non-hydroxylated and hydroxylated HIF1α-CODD peptides and integrating the peak area using RapidFire Integrator software (Agilent) (See Supplementary Methods for details).

**
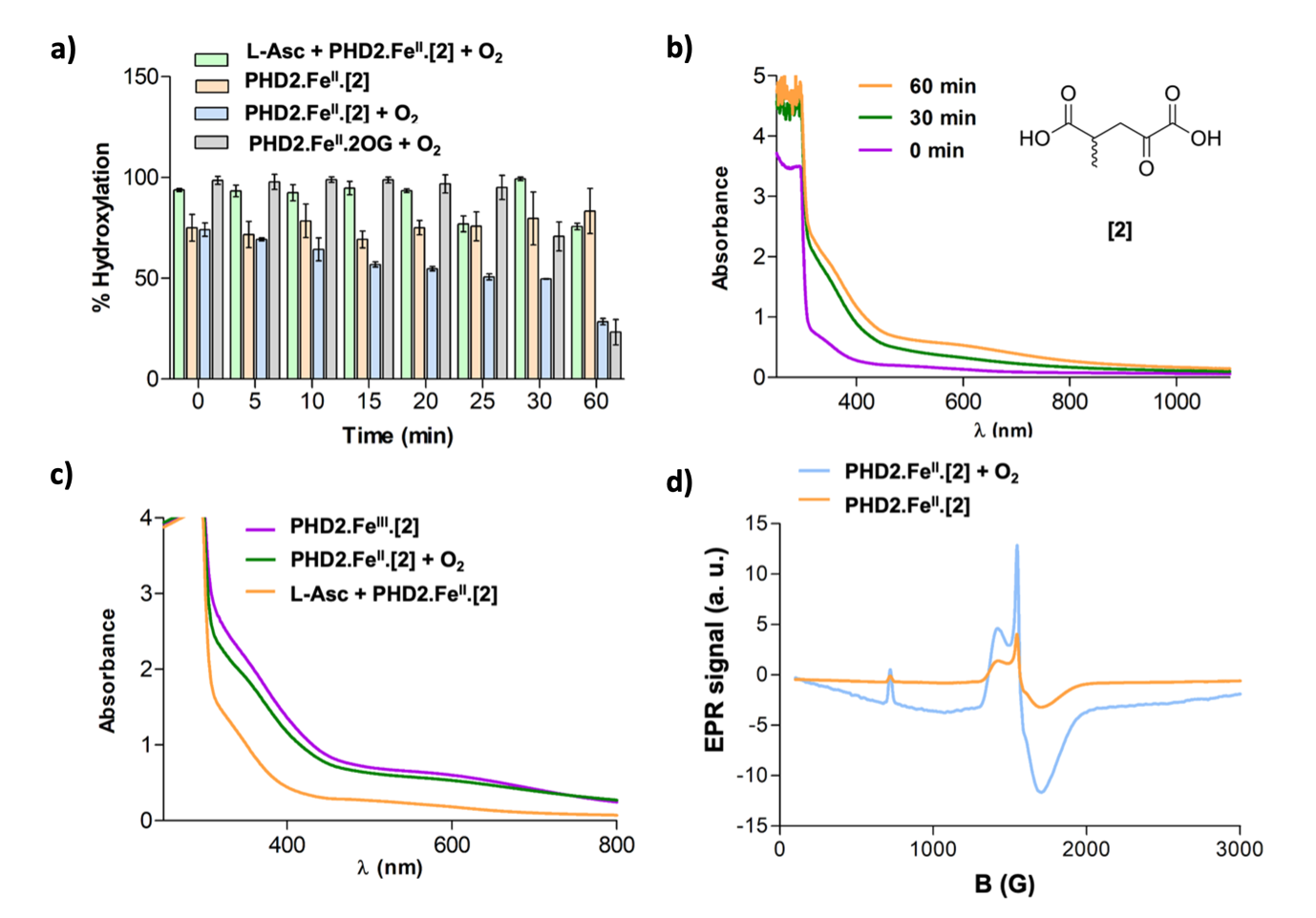
**

Figure S10. Hydroxylation assays and UV-vis spectra investigating the effects of O_2_ exposure on the PHD2.Fe(II).[2] complex. (a) The extent of hydroxylation observed when a PHD2.Fe(II).[2] mixture is added to an HIF1α-CODD_556-574_ solution, with or without prior O_2_ exposure of the PHD2.Fe(II).[2] mixture (blue and orange bars), and with L-Asc added to the PHD2.Fe(II).[2] mixture prior to O_2_ exposure (green bars). Grey bars: The extent of hydroxylation when a PHD2.Fe(II).2OG mixture is added to an HIF1α-CODD_556-574_ solution, with prior O_2_ exposure of the PHD2.Fe(II).2OG mixture. The results are the means of 3 independent repeats (n= 3, mean ± SD). (b) Time-course analysis of the UV-vis spectra of a mixture of 300 µM PHD2, 250 µM (NH_4_)_2_Fe(II)(SO_4_), 3 mM [2] exposed to O_2_ (c) Comparison of the UV-vis spectra of PHD2.Fe(II).[2] (green), PHD2.Fe(III).[2] (purple) and PHD2.Fe(II).[2] in the presence of 1 mM L-Asc (orange). (d) EPR spectra of anaerobic PHD2.Fe(II).[2] (orange) and O_2_ exposed (60 minutes) PHD2.Fe(II).[2] (blue). % hydroxylation is defined in the Supplementary Methods and was calculated extracting the data for the +2 charge states of the non-hydroxylated and hydroxylated HIF1α-CODD peptides and integrating the peak area using RapidFire Integrator software (Agilent) (See Supplementary Methods for details).


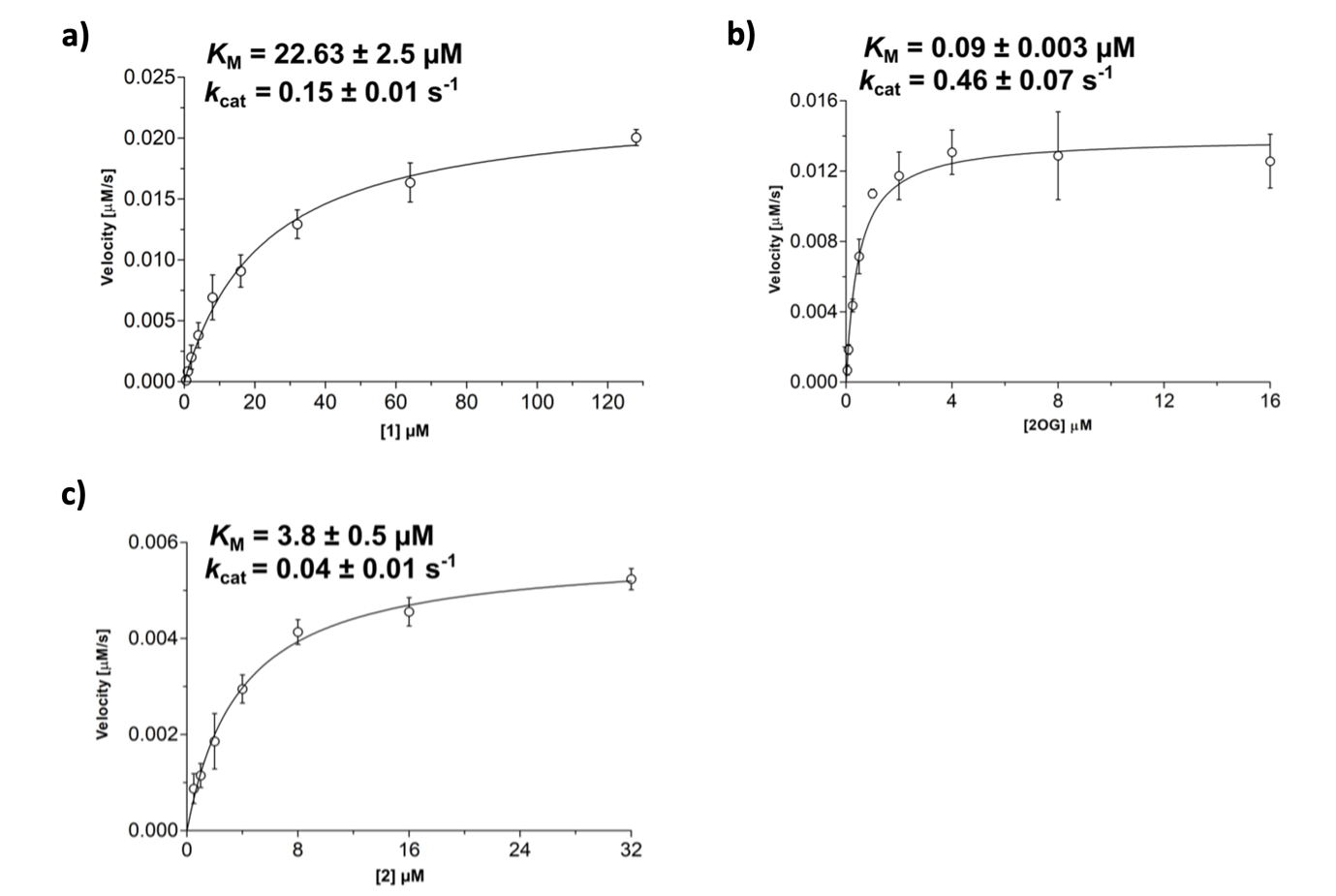


Figure S11. Steady-state kinetics at different concentration of 3-methyl-2OG, 2OG and 4-methyl-2OG (n=3). (a-c) Steady state kinetics of PHD2_181-407_ at different concentrations of 3-methyl-2OG [1], 2-oxoglutarate and 4-methyl-2OG [2], respectively. Conditions: 150 nM PHD2_181-407_, 20 µM (NH_4_)_2_Fe(II)(SO_4_), 100 µM L-Asc, 10 µM HIF1α-CODD_556-574_ and 0-100 µM [1]/[2]/2OG. The results are means of 3 independent repeats (n= 3, mean ± SD).


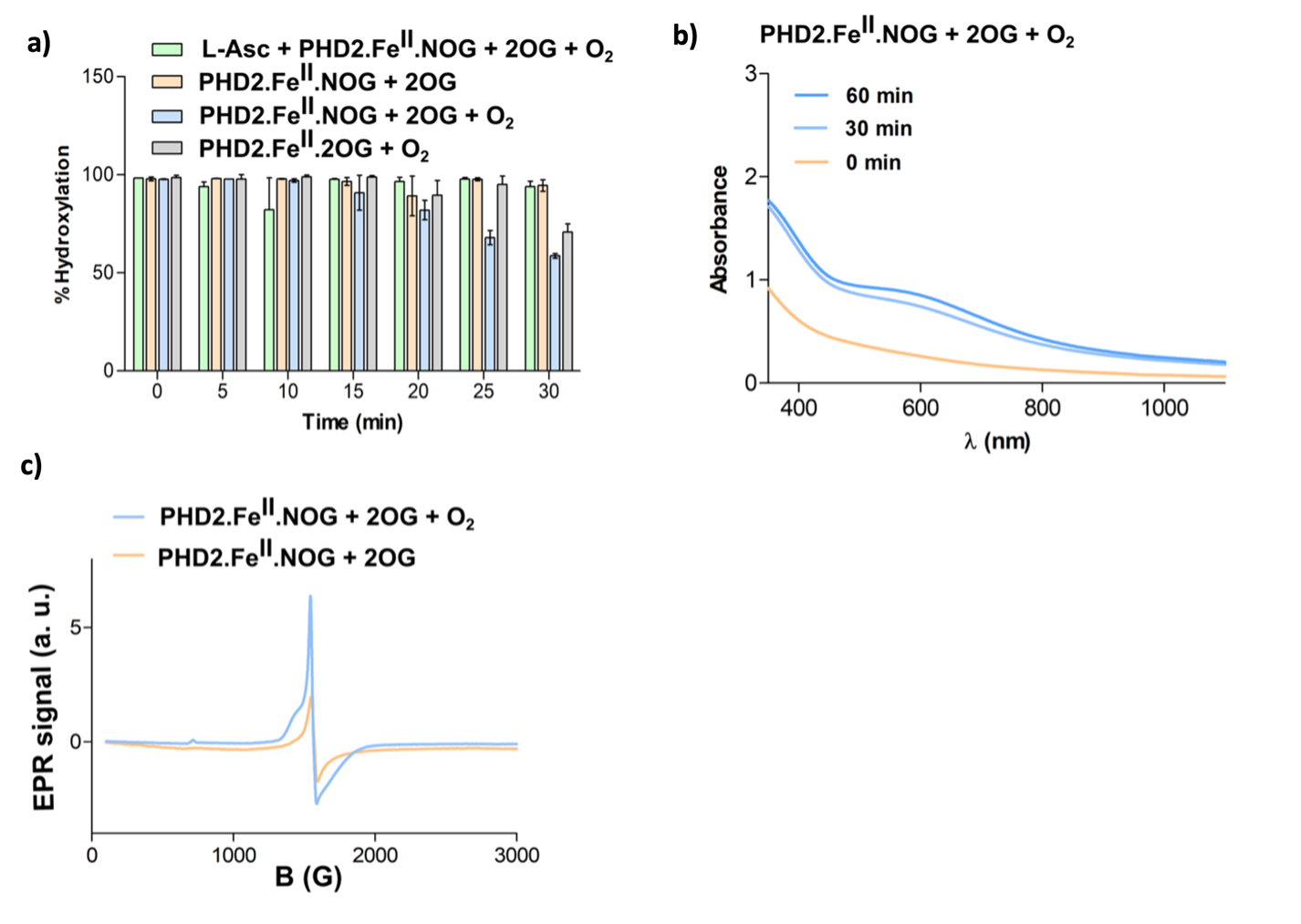


Figure S12. Hydroxylation assays and UV-vis spectra investigating the effects of O_2_ exposure on the mixture containing PHD2, Fe(II), NOG and 2OG. (a) The extent of hydroxylation when a PHD2.Fe(II).NOG(+2OG) mixture is added to an HIF1α-CODD_556-574_ solution, with and without prior O_2_ exposure of the PHD2.Fe(II).NOG(+2OG) mixture (blue and orange bars), with L-Asc added to the a PHD2.Fe(II).NOG(+2OG) mixture prior to O_2_ exposure (green bars). Grey bars: The extent of hydroxylation when a PHD2.Fe(II).2OG mixture is added to an HIF1α-CODD_556-574_ solution, with prior O_2_ exposure of the PHD2.Fe(II).2OG mixture. The results are means of 3 independent repeats (n= 3, mean ± SD). (b) Time-course of the UV-vis spectra of PHD2 (300 μM), (NH_4_)_2_Fe(II)(SO_4_) (250 μM), NOG (3 mM) and 2OG (3 mM) prior exposure to O_2_ (orange line) and after exposure to O_2_ (cyan and blue lines). (c) EPR spectra of anaerobic PHD2.Fe(II).NOG + 2OG (orange) and O_2_ exposed (60 minutes) (blue). The % hydroxylation is defined in the Supplementary Methods and was obtained extracting the data for the +2 charge states of the non-hydroxylated and hydroxylated HIF1α-CODD peptides and integrating the peak area using RapidFire Integrator software (Agilent) (See Supplementary Methods for details).


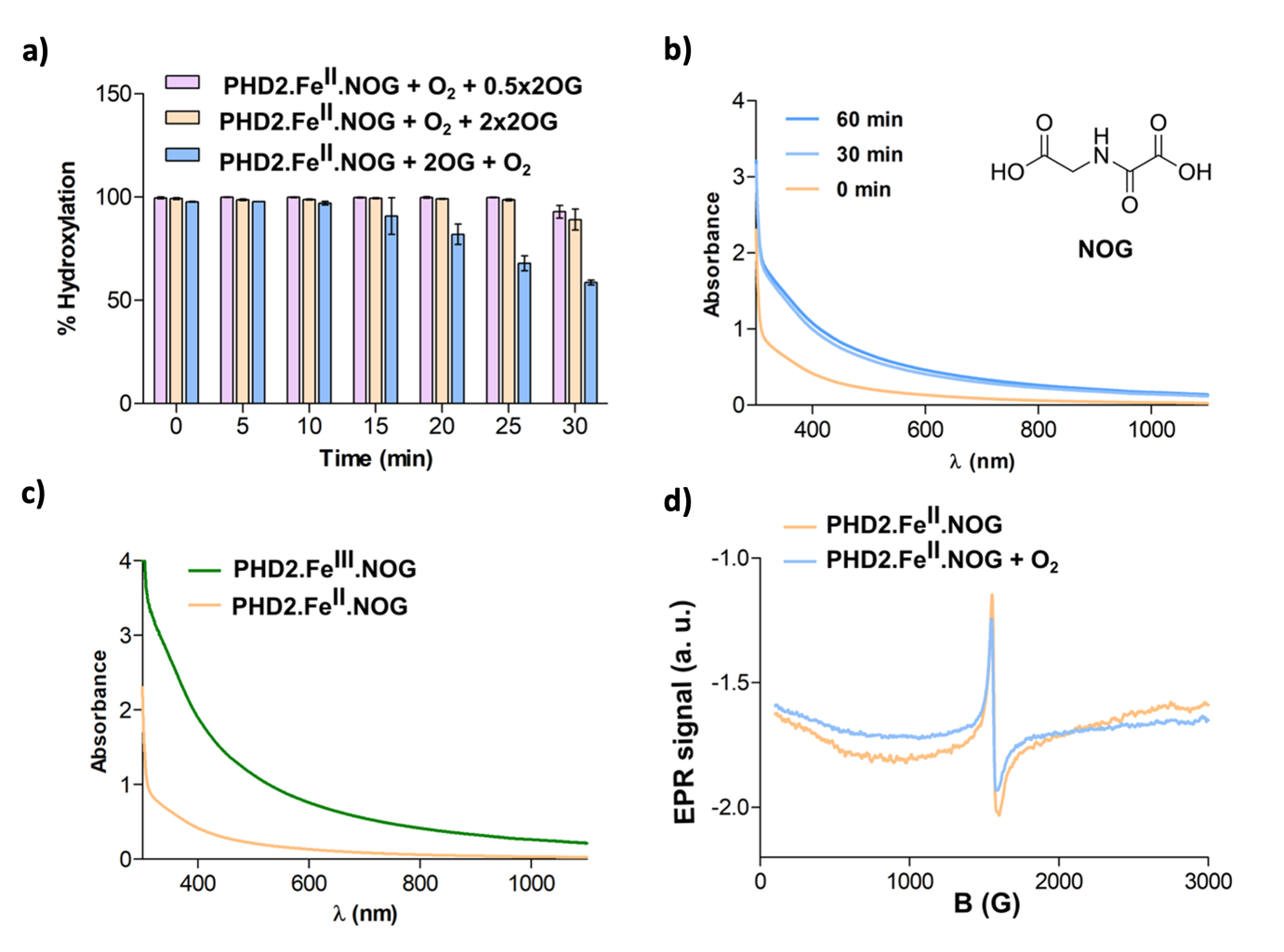


Figure S13. Hydroxylation assays and UV-vis spectra investigating the effects of O_2_ exposure on the PHD2.Fe(II).NOG complex. (a) The extent of hydroxylation when a PHD2.Fe(II).NOG mixture is added to an HIF1α-CODD_556-574_ and 2OG solution, with prior O_2_ exposure of the PHD2.Fe(II).NOG mixture (pink and orange bars). 2OG:NOG ratio of 1:2 (pink bars) and of 2:1 (orange bars). Blue bars: The extent of hydroxylation when a PHD2.Fe(II).NOG(+2OG) mixture is added to an HIF1α-CODD_556-574_ solution, with prior O_2_ exposure of the PHD2.Fe(II).NOG(+2OG) mixture. The results are means of 3 independent repeats (n= 3, mean ± SD). (b) Time-course of the UV-vis spectra of PHD2 (300 μM), (NH_4_)_2_Fe(II)(SO_4_) (250 μM), NOG (3 mM) prior exposure to O_2_ (orange line) and after exposure to O_2_ (cyan and blue lines). (c) Comparison of the UV-vis spectra of anaerobic PHD2.Fe(II).NOG (orange) and PHD2.Fe(III).NOG (green). (d) EPR spectra of anaerobic PHD2.Fe(II).NOG (orange) and O_2_ exposed (60 minutes) (blue). The % hydroxylation is defined in the Supplementary Methods and was obtained by extracting the data for the +2 charge state of the non-hydroxylated and hydroxylated HIF1α-CODD peptides and integrating the peak area using RapidFire Integrator software (Agilent) (See Supplementary Methods for details).

**
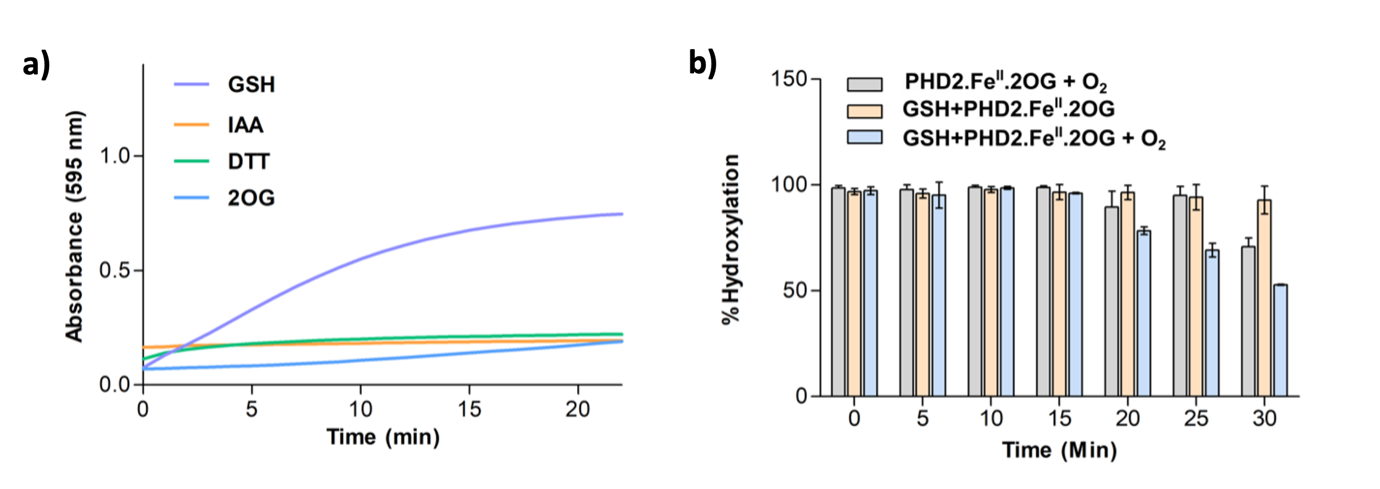
**

Figure S14. Rates of formation of the PHD2.Fe(III).2OG complex in the presence of different reducing agents. (a) Comparison of the rates of formation of the blue chromophore when 1 mM D-isoascorbic acid (orange), DTT (green) or GSH (violet) are present. (b) Comparison of the extents of hydroxylation observed on adding a GSH.PHD2.Fe(II).2OG mixture that was either anaerobic (orange bars) or which had been exposed to O_2_ (blue bars) to HIF1α-CODD_556-574_ after 0, 5, 10, 15, 20, 25, 30, and 60 minutes of incubation. Assays were conducted as described in the Supplementary Methods. Results are means of 3 independent repeats (n= 3, mean ± SD). The % hydroxylation is defined in the Experimental Procedures and was obtained extracting the data for the +2 charge state of the non-hydroxylated and hydroxylated HIF1α-CODD peptides and integrating the peak area using RapidFire Integrator software (Agilent) (See Supplementary Methods for details). DTT: dithiothreitol, GSH: glutathione.


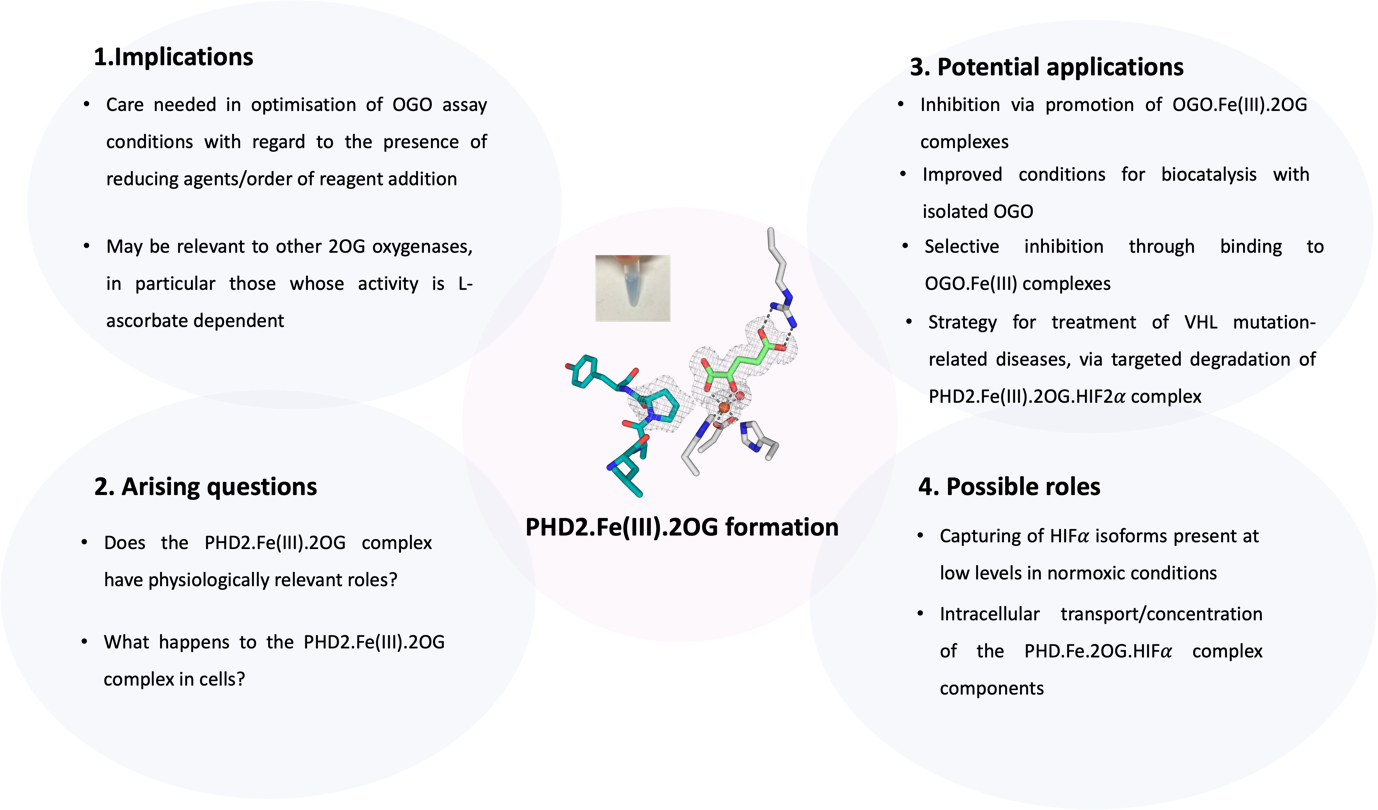


# Figure S15. Summary of the implications, questions arising, potential applications and roles linked to the observation of stable PHD2.Fe(III).2OG.(+/- HIFα) complexes.

Table S1. Data collection and refinement statistics for the PHD2_181-407_ crystal structures**.** Single crystal diffraction data were collected from samples at 100 K with conventional, rotation-based methods. Statistics for the highest-resolution shell are in parentheses.

|  | **PHD2_181-407_.ACT.HIF2α-CODD_523-542_** | **PHD2_181-407_.Fe(II).2OG.HIF2α-CODD_523-542_** | **PHD2_181-407_.Fe(III).2OG.HIF2α-CODD_523-542_** | **PHD2_181-407_.ACT.HIF2α-CODD_523-542_(OH)** |
| --- | --- | --- | --- | --- |
| **PDB** | 8Q5S | 8Q6D | 8Q6E | 8Q64 |
| **Wavelength (Å)** | 0.9762 | 0.9762 | 0.9762 | 0.9762 |
| **Resolution range** | 40.7 - 1.49 (1.543 - 1.49) | 35.71 - 1.4 (1.45 - 1.4) | 40.65 - 1.37 (1.419 - 1.37) | 42.47 - 1.36 (1.409 - 1.36) |
| **Space group** | *P*2_1_22_1_ | *P*2_1_22_1_ | *P*2_1_2_1_2 | *P*2_1_22_1_ |
| **Unit cell** | 37.8877 42.8242 130.807 90 90 90 | 38.07 42.63 130.75 90 90 90 | 130.9 38.12 42.77 90 90 90 | 38.0541 42.4674 130.457 90 90 90 |
| **Total reflections** | 448799 (45357) | 538982 (37894) | 91911 (8998) | 609453 (58409) |
| **Unique reflections** | 35727 (3518) | 42593 (3907) | 45973 (4514) | 46406 (4554) |
| **Multiplicity** | 12.6 (12.9) | 12.7 (9.7) | 2.0 (2.0) | 13.1 (12.8) |
| **Completeness (%)** | 96.98 (70.18) | 99.28 (92.90) | 99.77 (98.05) | 99.90 (99.61) |
| **Mean I/sigma(I)** | 8.52 (0.34) | 19.75 (2.97) | 14.12 (0.61) | 8.59 (0.45) |
| **Wilson B-factor** | 21.84 | 17.23 | 18.38 | 15.85 |
| **R-merge** | 0.1387 (3.304) | 0.06476 (0.7929) | 0.0249 (1.131) | 0.1727 (3.162) |
| **R-meas** | 0.1446 (3.44) | 0.06755 (0.837) | 0.03521 (1.6) | 0.1798 (3.295) |
| **R-pim** | 0.04043 (0.9484) | 0.0189 (0.2608) | 0.0249 (1.131) | 0.0494 (0.9188) |
| **CC1/2** | 0.999 (0.366) | 0.999 (0.872) | 1 (0.403) | 0.999 (0.379) |
| **CC*** | 1 (0.732) | 1 (0.965) | 1 (0.758) | 1 (0.741) |
| **Reflections used in refinement** | 34653 (2471) | 42584 (3901) | 45877 (4435) | 46368 (4544) |
| **Reflections used for R-free** | 1768 (114) | 2097 (190) | 2299 (263) | 2273 (198) |
| **R-work** | 0.1886 (0.3977) | 0.1670 (0.2412) | 0.1797 (0.4141) | 0.1906 (0.3932) |
| **R-free** | 0.2125 (0.3765) | 0.1900 (0.2468) | 0.2054 (0.4267) | 0.2120 (0.3799) |
| **CC(work)** | 0.970 (0.521) | 0.967 (0.898) | 0.972 (0.666) | 0.975 (0.524) |
| **CC(free)** | 0.970 (0.506) | 0.959 (0.837) | 0.953 (0.765) | 0.971 (0.396) |
| **Number of non-hydrogen atoms** | 2102 | 2140 | 2161 | 2177 |
| **macromolecules** | 1899 | 1895 | 1899 | 1923 |
| **ligands** | 21 | 15 | 20 | 28 |
| **solvent** | 191 | 234 | 246 | 238 |
| **Protein residues** | 239 | 240 | 238 | 239 |
| **RMS(bonds)** | 0.010 | 0.005 | 0.013 | 0.005 |
| **RMS(angles)** | 1.01 | 0.90 | 1.26 | 0.87 |
| **Ramachandran favored (%)** | 97.87 | 96.61 | 97.01 | 96.55 |
| **Ramachandran allowed (%)** | 2.13 | 2.97 | 2.99 | 3.45 |
| **Ramachandran outliers (%)** | 0.00 | 0.42 | 0.00 | 0.00 |
| **Rotamer outliers (%)** | 0.53 | 0.00 | 0.53 | 0.52 |
| **Clashscore** | 2.42 | 1.62 | 1.35 | 2.90 |
| **Average B-factor** | 29.46 | 23.84 | 25.52 | 23.31 |
| **macromolecules** | 28.49 | 22.74 | 24.36 | 22.08 |
| **ligands** | 44.16 | 14.89 | 20.47 | 35.03 |
| **solvent** | 38.16 | 33.17 | 34.74 | 32.45 |
| **Number of TLS groups** | 9 | 10 | 9 | 12 |
|  |  |  |  |  |

# Supplementary Methods

## Protein production and purification

Recombinant PHD2_181-407_ was produced in BL21 (DE3) *Escherichia coli* (New England Biolabs) cells as reported^9,10^, with minor modifications. Cells were grown (37 °C, 180 rpm), until an OD_600nm_ of ~0.8 was reached. Expression was induced by addition of 0.5 mM (final concentration) isopropyl-β-D-thiogalactopyranoside (IPTG) at 28 °C, followed by incubation for 5h prior to harvesting by centrifugation (5,000 x g, 10 minutes). Cells were frozen at -80 °C prior to use. Cell pellets were thawed at 4 °C in Lysis Buffer (20 mM TrisHCl pH 7.5, 0.5 M NaCl, 5 mM imidazole, 5% _(v/v)_ glycerol) supplemented with DNAseI and protease inhibitor tablets (Roche), then lysed using sonication (10 min elapsed time, 3 seconds on/off pulse). The obtained cell lysates were pelleted using a Beckman Coulter-Avanti-JHC centrifuge (48,254 g, 30 minutes). The supernatant was loaded onto a 5 mL HisTrap™ column (Cytiva Life Sciences). Protein was eluted with Elution Buffer (20 mM TrisHCl pH 7.5, 0.5 M NaCl, 500 mM imidazole, 5%_(v/v)_ glycerol) using a step-gradient elution method (16%_(v/v)_, 34%_(v/v)_ and 100%_(v/v)_ Elution buffer, each 5CV). Fraction purity was analysed by SDS-PAGE. Fractions containing highly purified protein were concentrated using 10k MWCO Amicon® Ultra-15 machine (Merck Millipore). 0.25 units of 1X thrombin (Novagen, Merck) and 1X thrombin Cleavage Buffer (10X stock of 200 mM Tris-HCl pH 8.4, 1.5 M NaCl, and 25 mM CaCl_2_, Novagen, Merck) were added to the fractions to cleave the His_6_-tag. 20 mM EDTA pH 7.5 was added to chelate metal ions for 16 hours. Cleaved apo-PHD2_181-407_ was further purified by size-exclusion chromatography (Superdex-75 16/60 column (GE Healthcare)) using 50 mM Tris-HCl pH 7.5 RT, 1%_(v/v)_ glycerol as buffer.

## Solid-phase extraction-MS activity assays

*Steady-state kinetics assays*

Real-time solid-phase extraction (SPE)-MS assays were performed in Reaction Buffer (50 mM Tris-HCl pH 7.8, and 150 mM NaCl) made up in water (LC-MS Grade, LiChrosolv^®^). 100‍ mM stock solution of sodium-L-ascorbate, 2OG and 2OG analogues were made in water (LC-MS Grade, LiChrosolv^®^). A 10 mM HIF1α-CODD_556-574_ stock solution was made up in DMSO (>99%). 100 mM stock solution of (NH_4_)_2_Fe(II)(SO_4_) was made up in 20 mM HCl and then diluted to 1 mM using water (LC-MS Grade, LiChrosolv^®^). Assay conditions were as reported, with some modifications ^9,10^. 1 mL substrate mixture solutions containing 200 µM sodium-L-ascorbate, 20 µM (NH_4_)_2_Fe(II)(SO_4_), 10 µM HIF1α_556-574_-CODD and different 2OG/2OG analogues concentrations (0-120 µM) were prepared for the no-enzyme control reaction. 500 µL of the substrate mixture was transferred in 96-well polypropylene plates (Agilent). 500 µL of 300 nM PHD2_181-407_ in the Reaction Buffer was added to each well to initiate reaction; the first injection onto the C4 SPE cartridge (Agilent) was without enzyme (no-enzyme control).

*Activity assays for PHD2.Fe(III).2OG complexes*

Mixtures of 300 µM PHD2_181-407_, 250 µM Fe(II)SO_4_(NH_4_)_2_ and 3 mM 2OG,3-methyl-2OG (**1**) or 4-methyl-2OG (**2**) were prepared in an anaerobic chamber. Each mixture was split in to two aliquots, one of which was removed from the anaerobic box and exposed to O_2_. After 5, 10, 15, 25, 30 and 60 minutes of O_2_ exposure, 1 µL of the PHD2.Fe.2OG/**1**/**2** mixture was added to 100 µL of the Reaction Buffer containing HIF1α-CODD_556-574_ 10 µM. Reactions were incubated for 30 minutes, then quenched with 10 µL of aqueous formic acid (0.1%_(v/v)_). For anaerobic controls, 1 µL of the anoxic PHD2.Fe(II).2OG/**1**/**2** mixture was added to 100 µL of aerobic Reaction Buffer containing HIF1α-CODD_556-574_ 10 µM after 5, 10, 15, 25, 30 and 60 minutes of being made. As before, the reaction was incubated for 30 minutes before quenching with 0.1%_(v/v)_ formic acid. The same experiment was conducted adding L-Asc (1 mM) or GSH (1 mM) in the starting PHD2.Fe(II).2OG/**1**/**2** mixture prior to O_2_ exposure and substrate addition. Note that assays to assess the activity changes of solutions of PHD2.Fe(II) and PHD2.Fe(II).NOG complexes were conducted with some variations from that described above, that is: 1 µL of a PHD2.Fe(II) or PHD2.Fe(II).NOG mixture was added to 100 µL of the Reaction Buffer containing HIF1α-COOD_556-574_ 10 µM and 2OG (3 mM) for both anaerobic control and O_2_ exposed samples.

A RapidFire^®^ RF360 sampling robot (Agilent Technologies, Inc.) was used to monitor peptide-hydroxylation as reported^9,10^. The data were extracted for the +2 charge state of the peptide and RapidFire Integrator software (Agilent) was used to integrate the peak area. The % conversion of the HIF1α-CODD peptide to the +16 hydroxylated peptide was calculated using: % hydroxylated substrate = 100 x hydroxylated/(hydroxylated + non-hydroxylated). Data were normalized to no enzyme control. GraphPad Prism 5 was used to display the data and measure K_M_ and *k*_cat_ values using the Nonlinear Regression fitting tools.

**Protein LC-MS assays**

Protein LC-MS spectra were collected on apo-PHD2_181-407_, anaerobic and O_2_ exposed PHD2_181-407_.Fe(II).2OG samples. The apo-PHD2_181-407_ sample was prepared by diluting 300 µM PHD2 in Reaction Buffer (50 mM Tris-HCl pH 7.8, and 150 mM NaCl) to a final concentration of 20 µM. Anaerobic PHD2_181-407_.Fe(II).2OG was prepared by mixing 300 µM PHD2, 300 µM Fe(II) and 3 mM 2OG in an anaerobic chamber; the mixture was then diluted tenfold 1:10 using the Reaction Buffer. 10 µL of formic acid (10 % _v/v_) were added to the anaerobic PHD2_181-407_.Fe(II).2OG sample prior to data collection. The O_2_ exposed PHD2_181-407_.Fe(II).2OG sample was prepared mixing 300 µM PHD2, 300 µM Fe(II) and 3 mM 2OG in an aerobic environment. 10 µL of formic acid (10 % _v/v_) was added to the solution after 60 minutes (note that at this point the solution was visibly blue).

Samples were injected into a Waters Xevo-G2-S QTOF mass spectrometer equipped with a ProSwift RP-1S phenyl LC column (ThermoFisher Scientific Inc.). Milli-Q water containing 0.1% _(v/v)_ formic acid (Solvent A) and acetonitrile with 0.1% _(v/v)_ formic acid (Solvent B) were used to elute the samples with a gradient-elution method (from 5% _(v/v)_ Solvent B to 95% _(v/v)_ Solvent B). The resultant data were analysed and deconvoluted using MassLynx.

## ^1^H NMR assays

PHD2_181-407_ was buffer exchanged into Tris-d11-DCl buffer (pD 7.5, 50 mM in D_2_O). Stock solutions of 2OG and (+)-sodium-L-ascorbate (L-Asc) were made up in D_2_O and a stock solution of Fe(II)SO_4_(NH_4_)_2_ was made up in 20 mM DCl prior to each experiment. Reactions were conducted in 5 mm diameter NMR tubes at 310 K and initiated by 2OG addition at the following final concentrations: 20 µM PHD2_181-407_, 50 µM Fe(II), 1 mM 2OG and 4 mM L-Asc. A Bruker AVIII 700 machine was used to acquire ^1^H NMR spectra. Spectra were collected in 75 s intervals and the D_2_O signal (4.72 ppm) was used as an internal lock signal. Peaks were integrated using MestReNova as reported ^8,11^.

## Crystallography

PHD2_181-407_.ACT.HIF2αCODD crystals were obtained from a starting solution containing 1 mM PHD2, 0.8 mM (NH_4_)_2_Fe(II)(SO_4_), 3 mM 2OG and 1 mM HIF2α-CODD_523-542_. The concentration of 2OG in solution was increased to obtained the other reported structures, for which the starting mixture contained: 1 mM PHD2_181-407_, 1 mM (NH_4_)_2_Fe(II)(SO_4_), 6 mM 2OG and 1 mM HIF2α-CODD_523-542_. Note that to obtain the PHD2_181-407_.Fe(III).2OG.HIF2α-CODD_523-542_ crystals, HIF2α-CODD_523-542_ was added after the starting PHD2.Fe(II).2OG solution mixture has become visibly blue (**Fig S3.b**). Mixtures were dispensed in a 1:1 ratio of protein to precipitant in a 24-well sitting drop plate. The precipitant was a mixture of (10-35%) PEG 4K, 0.2 M ammonium acetate and 0.1 M sodium ammonium acetate trihydrate pH (4.1-5.6). Crystals were cryo-protected with 10%_(v/v)_ DMSO (>99%) in the precipitant solution and flash-frozen in liquid N_2_. Diffraction data were collected at the Diamond Light Source beamline i03 (proposal MX-23459), employing automated data collection. 3,600 images at 0.1° rotations were collected (wavelength: 0.9762 Å)**.** See **Table S1** for data collection/refinement statistics.

## Crystallographic data processing

Data were auto-processed with DIALS using Xia2^12^. Phenix^13^ was used to refine models; PHENIX.Xtriage^14^ was used to assess data quality. Structures were solved using PHASER-MR^15^, employing PDB: **7Q5X** as a search model^9^. Models were refined using PHENIX.refine^14,16^ with iterative manual model building using COOT^17,18^. The Research Collaboratory for Structural Bioinformatics PDB tool was used to prepare coordinate and structure factors files in mmCIF format for deposition ^19^. PyMOL^™^ (Schrodinger) was used for graphical representation and structural superimpositions.

**References**

1. Chowdhury, R. *et al.* Structural basis for oxygen degradation domain selectivity of the HIF prolyl hydroxylases. *Nat Commun* **7**, 1–10 (2016).

2. Domene, C., Jorgensen, C. & Schofield, C. J. Mechanism of Molecular Oxygen Diffusion in a Hypoxia-Sensing Prolyl Hydroxylase Using Multiscale Simulation. *J Am Chem Soc* **142**, 2253–2263 (2020).

3. Hausinger, R. P. Fe(II)/α-Ketoglutarate-Dependent Hydroxylases and Related Enzymes. *Crit Rev Biochem Mol Biol* **39**, 21–68 (2004).

4. Krebs, C., Fujimori, D. G., Walsh, C. T. & Bollinger, J. M. Non-heme Fe(IV)-oxo intermediates. *Acc Chem Res* **40**, 484–492 (2007).

5. Mitchell, A. J. *et al.* Visualizing the reaction cycle in an Iron(II)- and 2-(Oxo)-glutarate-dependent hydroxylase. *J Am Chem Soc* **139**, 13830–13836 (2017).

6. Elkins, J. M. *et al.* Structure of Factor-inhibiting Hypoxia-inducible Factor (HIF) Reveals Mechanism of Oxidative Modification of HIF-1α. *Journal of Biological Chemistry* **278**, 1802–1806 (2003).

7. Hangasky, J. A., Gandhi, H., Valliere, M. A., Ostrom, N. E. & Knapp, M. J. The rate-limiting step of O2 activation in the α-ketoglutarate oxygenase factor inhibiting hypoxia inducible factor. *Biochemistry* **53**, 8077–8084 (2014).

8. Flashman, E. *et al.* Evidence for the slow reaction of hypoxia-inducible factor prolyl hydroxylase 2 with oxygen. *FEBS J* **277**, 4089–4099 (2010).

9. Figg, W. D. *et al.* Structural basis for binding of the renal carcinoma target hypoxia-inducible factor 2α to prolyl hydroxylase domain 2. *Proteins: Structure, Function, and Bioinformatics* **91**, 1510–1524 (2023).

10. Holt-Martyn, J. P. *et al.* Structure-Activity Relationship and Crystallographic Studies on 4-Hydroxypyrimidine HIF Prolyl Hydroxylase Domain Inhibitors. *ChemMedChem* **15**, 270–273 (2020).

11. Tarhonskaya, H. *et al.* Investigating the contribution of the active site environment to the slow reaction of hypoxia-inducible factor prolyl hydroxylase domain 2 with oxygen. *Biochemical Journal* **463**, 363–372 (2014).

12. Winter, G. *et al.* DIALS: implementation and evaluation of a new integration package. *Acta Crystallogr D Struct Biol* **74**, 85–97 (2018).

13. Liebschner, D. *et al.* Macromolecular structure determination using X-rays, neutrons and electrons: recent developments in Phenix. *Acta Crystallogr D Struct Biol* **75**, 861 (2019).

14. Adams, P. D. *et al.* PHENIX: a comprehensive Python-based system for macromolecular structure solution. *Acta Crystallogr D Biol Crystallogr* **66**, 213–221 (2010).

15. McCoy, A. J. *et al.* Phaser crystallographic software. *J Appl Crystallogr* **40**, 658–674 (2007).

16. Afonine, P. V. *et al.* Towards automated crystallographic structure refinement with phenix.refine. *Acta Crystallogr D Biol Crystallogr* **68**, 352–367 (2012).

17. Emsley, P., Lohkamp, B., Scott, W. G. & Cowtan, K. Features and development of Coot. *Acta Crystallogr D Biol Crystallogr* **66**, 486–501 (2010).

18. Winn, M. D. *et al.* Overview of the CCP4 suite and current developments. *Acta Crystallogr D Biol Crystallogr* **67**, 235–242 (2011).

19. Young, J. Y. *et al.* OneDep: Unified wwPDB System for Deposition, Biocuration, and Validation of Macromolecular Structures in the PDB Archive. *Structure* **25**, 536–545 (2017).
